# *Solanum nigrum* Linn.: An Insight into Current Research on Traditional Uses, Phytochemistry, and Pharmacology

**DOI:** 10.3389/fphar.2022.918071

**Published:** 2022-08-16

**Authors:** Xufei Chen, Xufen Dai, Yinghai Liu, Yan Yang, Libang Yuan, Xirui He, Gu Gong

**Affiliations:** ^1^ Department of Anesthesiology, The General Hospital of the Western Theater Command, Chengdu, China; ^2^ Shaanxi Institute for Food and Drug Control, Xi’an, China; ^3^ Department of Bioengineering, Zhuhai Campus, Zunyi Medical University, Zhuhai, China

**Keywords:** traditional use, phytochemistry, pharmacology, toxicology Taylor and Francis, *Solanum nigrum Linn.*

## Abstract

*Solanum nigrum* Linn., is a common edible medicinal herb of the Solanaceae family which is native to Southeast Asia and is now widely distributed in temperate to tropical regions of Europe, Asia, and America. Traditionally, it has been used to treat various cancers, acute nephritis, urethritis, leucorrhea, sore throat, toothache, dermatitis, eczema, carbuncles, and furuncles. Up to now, 188 chemical constituents have been identified from *S. nigrum*. Among them, steroidal saponins, alkaloids, phenols, and polysaccharides are the major bioactive constituents. Investigations of pharmacological activities of *S. nigrum* revealed that this edible medicinal herb exhibits a wide range of therapeutic potential, including antitumor, anti-inflammatory, antioxidant, antibacterial, and neuroprotective activities both *in vivo* and *in vitro*. This article presents a comprehensive and systematic overview of the botanical, traditional uses, phytochemical compositions, pharmacological properties, clinical trials, and toxicity of *S. nigrum* to provide the latest information for further exploitation and applications of *S. nigrum* in functional foods and medicines.

## Graphical Abstract

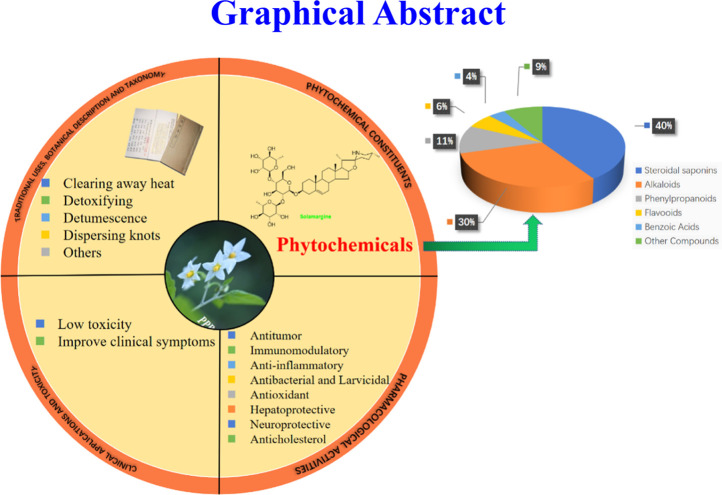

## Introduction

**FIGURE 1:**
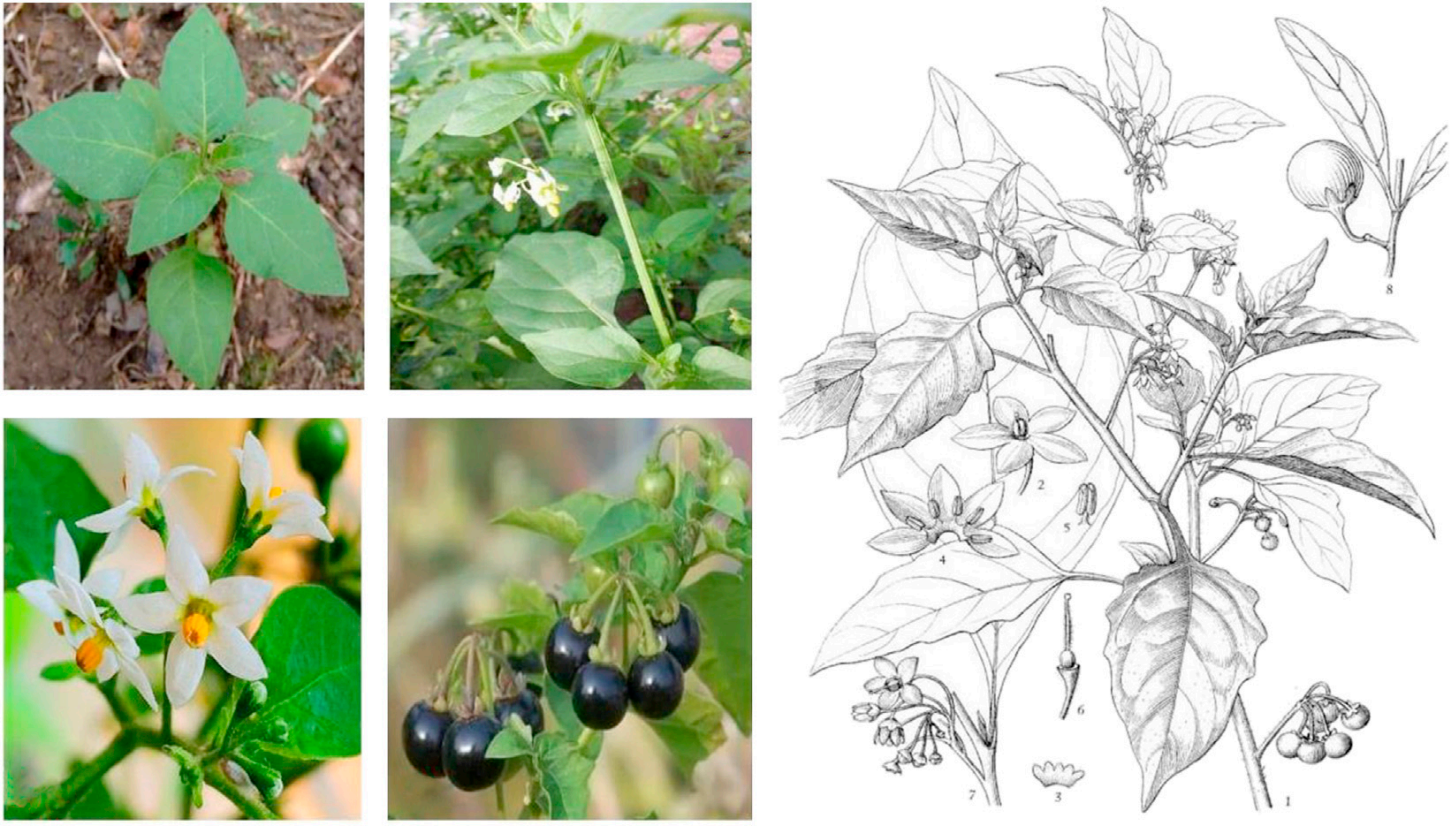
Leaves **(A)**; stems and leaves **(B)**; flowers **(C)**; fruits **(D)**; illustration of *S. nigrum* **(E)** (1, Upper portion of plant with flowers and fruits; 2, Flower; 3, Opened calyx adaxial view; 4, Opened corolla showing stamens; 5, Stamen; 6, Pistil; 7, Flowering branch; 8, Fruiting branch).

The genus *Solanum* (Solanaceae family) consists of more than 2,000 species, which are distributed worldwide in tropical and subtropical regions. They mostly have beautiful flowers and fruits. In China, 39 species and 14 varieties of *Solanum* exist (Editorial Committee of Flora of China, 1979). *Solanum nigrum* Linn. (Figure 1), also known as *Solanum nigrum* var*. virginicum* L*.* and “龙葵” (in Chinese). *S. nigrum* is distributed in almost every province in China and is commonly found near the fields, wastelands, and villages. It is also widely distributed in the temperate to tropical regions of Europe, Asia, and America (The Plant List, 2013; Xiang et al., 2019). In China, the plant is known under local names, such as “Yelahu”, “Yehaijiao”, “Heixingxing”, “Heitiantian”, “Kukui”, “Kucai”, “Heidoudou”, and “Yesanzi” (Flora of China, 2007).

*S. nigrum* can be used as a medicine and tastes bitter, is of cold property and slightly toxic, and belongs to the lung and kidney meridians. In Chinese folk medicine and traditional Chinese medicine (TCM), people have accumulated rich clinical experience in the use of *S. nigrum*. The whole plant of *S. nigrum* has good effects of dispersing blood stasis and detumescence, clearing away heat, as well as detoxification and has been commonly used for the treatment of canker sores, skin eczema, urinary tract infections, bacterial dysentery, prostate, and chronic bronchitis, etc. for thousands of years (Gao et al., 2021). In addition, in modern clinical practice, *S. nigrum* is commonly combined with other drugs for the treatment of cancers, such as lung cancer, cervical cancer, breast cancer, esophageal cancer, stomach cancer, liver cancer, and bladder cancer. In other Asian countries, such as Japan and India, it has also been documented for the treatment of tumors. Ripe berries of *S. nigrum* are sweet and salty and were reported to have been used as a famine food in China in the 15th century. In India, the leaves and berries of this plant are commonly consumed as food or vegetable after cooking (Wang, 2007; Zhao, 2010; Wang et al., 2017). In the past few decades, phytochemical research has confirmed that the whole *S. nigrum* herb contains steroidal saponins, steroidal alkaloids, flavonoids, coumarin, lignin, organic acids, volatile oils, polysaccharides, and other ingredients. The crude extract of *S. nigrum* and some of the above-mentioned compounds have been confirmed to have various effects, including antitumor, antioxidative, anti-inflammatory, hypotensive, neuroprotective, immunomodulatory, antibacterial, and liver protective effects. Especially the antitumor effect of steroidal saponins and steroidal alkaloids is a research hotspot, and drug researchers expect to find antitumor lead compounds from these components.

In view of the increasing interest in steroid derivatives obtained from *S. nigrum* due to their significant pharmacological activity, this review provides a systematic summary and criticism of the traditional uses, phytochemistry, pharmacological activity, and safety of *S. nigrum* based on the literature obtained from databases, with emphasis on the pharmacological activity and potential applications of *S. nigrum* in TCM. We believe that this review is of great significance for the further research or development of antioxidant functional foods, and novel antitumor drugs based on *S. nigrum* and its active compounds.

## Research Methodology

The literature of this review (until March 2022) was obtained from various important databases, such as Google Scholar, Baidu Scholar, Web of Science, SciFinder Scholar, PubMed, published classic texts of Chinese herbal medicines (e.g., *Sheng Ji Zong Lu*), the China Knowledge Resource Integrated Database from the China National Knowledge Infrastructure (CNKI), publications in peer-reviewed journals, Ph.D. and M.Sc. theses, as well as other web sources, such as Flora of China, the Plant List, and YaoZh website (https://db.yaozh.com). Keywords used in the literature search were: “*Solanum nigrum* Linn.”, “Long Kui/龙葵”, “phytochemistry”, “pharmacology”, “biological activity”, “traditional uses”, “secondary metabolites”, “safety”, “toxicology”, and “clinical trial”. After reviewing a total of 576 scientific publications on *S. nigrum*, excluding some irrelevant content, we mainly focused on 120 documents. ChemDraw Ultra 15.0 software was used to draw the chemical structures.

## Botanical Description and Taxonomy

### Botanical Description

*S. nigrum* is an annual erect herbaceous plant with 0.25–1 m in high. It has a taproot system with a well-developed main root and is often lignified. The stem has no inconspicuous edges, is green or purple in color, and nearly glabrous or puberulent. The leaf is ovate, 2.5–10 cm long, and 1.5–5.5 cm wide, and its apex is shortly pointed. The cuneate is base wedge shaped to broad and descending to the petiole, with irregular wavy coarse teeth throughout or on each side and smooth or sparse, soft, and hairy on both sides with five to six veins on each side, and the petiole is about 1–2 cm long. The scorpion-tailed inflorescence is extra-axillary and composed of 3-6-(10) flowers. The total pedicel is about 1–2.5 cm long, and the pedicel is about 5 mm long and nearly glabrous or pubescent. The calyx is small, shallow cup shaped, and about 1.5–2 mm in diameter, and the teeth are oval, the tip is round, and the junction between the two teeth at the base is angled. The corolla is white, the tube is hidden in the calyx and less than 1 mm in length, and the 5-parted crown is about 2.5 mm in length. The lobes are ovoid and about 2 mm long. The filaments are short, the anthers are yellow, about 1.2 mm long, and about four times the length of the filaments, and the apical hole is inward. The ovary is ovoid and about 0.5 mm in diameter, and the style is about 1.5 mm long. The lower part of the middle part is covered with white hairs, the stigma is small, and the head is shaped. The berry is spherical, about 8 mm in diameter, and black when ripe. The seeds are mostly nearly ovoid, about 1.5–2 mm in diameter, and compressed on both sides (Flora of China, 2007).

### Taxonomy

*S. nigrum* in botanical classification as Plantae, Angiospermae, Magnoliopsida, Solanales, Solanaceae, Solanum. Solanaceae is a family of plants with about 80 genera and 3,000 species, widely distributed in tropical and temperate regions, mainly in tropical America. Solanum occupies an important weight in the Solanaceae family, with about 2,000 species, among which the well-known varieties are *Solanum melongena* L., *Solanum tuberosum* L., *S. nigrum*, etc. (The Plant List, 2013; Flora of China, 2007). The spherical berries of *S. nigrum* are dark purple when ripe. Both the berries and the leaves are edible, but the leaves contain high amounts of alkaloids that must be cooked to detoxify.

## Traditional Uses

The first known record describing the medicinal use of *S. nigrum* was found in *Yao Xing Lun* (药性论, Tang Dynasty) (Editorial Committee of State Administration of traditional Chinese Medicine, 1999). Since then, the medicinal use of *S. nigrum* was increasingly reported in many other well-known classical TCM monographs, including *Xin Xiu Ben Cao* (新修本草, Tang Dynasty), *Shi Liao Ben Cao* (食疗本草, Tang Dynasty), *Ben Cao Tu Jing* (本草图经, Song Dynasty), *Jiu Huang Ben Cao* (救荒本草, Ming Dynasty), *Dian Nan Ben Cao* (滇南本草, Ming Dynasty), *Ben Cao Gang Mu* (本草纲目, Ming Dynasty), *Dian Nan Ben Cao Tu Shuo* (滇南本草图说, Ming Dynasty), and *Ben Cao Gang Mu Shi Yi* (本草纲目拾遗, Qing Dynasty). In these classical books of TCM, it is recorded that *S. nigrum* has the functions of clearing away heat, detoxification, detumescence, and dispersion of knots (Editorial Committee of Nanjing University of Chinese Medicine, 2006). In all of these major TCM monographs, *S. nigrum* has different medicinal properties, including the treatment of sore carbuncle swelling poison, skin eczema, poor urination, chronic bronchitis, excessive leucorrhea, prostatitis, and dysentery. Many TCM herbs or classical prescriptions containing *S. nigrum* have been used in the form of decoction, powders, granules, tablets, and pills. The traditional and modern prescriptions of *S. nigrum* commonly used in China are presented in Table 1. For example, according to *Sheng Ji Zong Lu*, *S. nigrum* (30 g) is compatible with other herbs such as *Hylotelephium erythrostictum* (Miq.) H. Ohba (30 g), *Coptis chinensis* Franch. (30 g), *Momordica cochinchinensis* (Lour.) Spreng. (15 g), and *Abelmoschus manihot* (Linn.) Medicus (15 g) for the treatment of malignant sores (https://db.yaozh.com). For the treatment of carbuncles, swelling, and poisoning, *S. nigrum* can be externally applied for washing and smashing. It can also be combined with TCM, such as *Corydalis bungeana* Turcz., *Chrysanthemum indicum* L., and *Taraxacum mongolicum* Hand.-Mazz., for decoction and subsequent oral administration to treat sore throats. In addition, *S. nigrum* has a diuretic effect and can be used together with *Alisma plantago-aquatica* Linn., *Akebia quinata* (Houttuyn) Decaisne, and other drugs to treat edema, adverse urination and other diseases (Editorial Committee of Nanjing University of Chinese Medicine, 2006). In recent years, it has been commonly used together with *Duchesnea indica* (Andr.) Focke, *Hedyotis diffusa* Willd., *Solanum lyratum* Thunberg, etc. in clinic for the treatment of cancer (Liu et al., 2019a).

**TABLE 1:** The traditional and modern prescriptions of *S. nigrum* in China.

Preparation name	Composition	Route of administration	Dosage form	Indications for use	References
Longkui san	***S. Nigrum***, *Hylotelephium erythrostictum* (Miq.) H. Ohba, *Coptis chinensis* Franch., *Os Draconis* (FossiliaOssiaMastodi), *Boswellia carterii* Birdw., *Momordica cochinchinensis* (Lour.) Spreng., *Abelmoschus manihot* (Linn.) Medicus	External use	Making into powder	Malignant sores	“*Sheng Ji Zong Lu*” (Song Dynasty, A.D. 1117)
Longkui san	***S. Nigrum***, *Boswellia carterii* Birdw., *Prunus armeniaca* L., *Coptis chinensis* Franch	External use	Making into powder	Malignant sores	“*Sheng Ji Zong Lu*” (Song Dynasty, A.D. 1117)
Longkuigeng san	***S. Nigrum***, *Moschus berezovskii* Flerov	External use	Making into powder	Back sore	“*Sheng Ji Zong Lu*” (Song Dynasty, A.D. 1117)
Longkui gao	***S. Nigrum***, *Hyoscyamus niger* Linn., *Allium sativum* L., *Coriandrum sativum* Linn., *Prunus armeniaca* L.	External use	Making into cream	Malignant prickling sore pain	“*Tai Ping Sheng Hui Fang*” (Song Dynasty, A.D. 992)
Xinlikang capsules	*Scutellaria barbata* D. Don, ***S. Nigrum***, *Duchesnea indica* (Andr.) Focke, *Astragalus membranaceus* (Fisch.)Bge*.*, *Panax ginseng* C. A. Meyer, *Saussurea involucrata* (Kar. et Kir.) Sch.-Bip*.*, *Angelica sinensis* (Oliv.) Diels, *Curcuma longa* Linn*.*, *Salvia miltiorrhiza* Bunge	Take orally	Decocting with water, and then making into granules	Invigorate qi and nourish blood, remove blood stasis and detoxify, cancer	Liu et al. (2019a)
Compound Tianxian capsules	*Trichosanthes kirilowii* Maxim., *Clematis chinensis* Osbeck, *Hedyotis diffusa* Willd., *Bos taurus domesticus* Gmelin, ***S. Nigrum***, *Arisaema heterophyllum Blume*, *Boswellia carterii* Birdw., *Commiphora myrrha* (Nees) Engl., *Panax ginseng* C. A. Meyer, *Astragalus membranaceus* (Fisch. )Bge., *Polyporus umbellatus* (Pers.) Fr.*, Elphe taeniura* Cope*, Cinnamomum camphora* (L. ) Presl, *Moschus berezovskii* Flervo	Take orally	Making into capsules	Supervened after esophageal cancer and stomach cancer, peptic ulcer	Kang et al. (2014)
Loulian capsules	*Hedyotis diffusa* Willd., *Semiaquilegia adoxoides* (DC.) Makino, *Polygonum onrientale* L., *Paris polyphylla* Smith. var. *yunnanensis* (Franch.) Hand.-Mzt., *Amyda sinensis* (Wiegmann), *Curcuma zedoaria* (Christm.) Rosc*.*, *Lobelia chinensis* Lour., *Steleophaga Plancyi* (Boleny), *Whitmania pigra* Whitman, *Panax ginseng* C. A. Meyer, *Fallopia multiflora* (Thunb.) Harald., ***S. Nigrum***	Take orally	Making into capsules	Hepatitis, liver cirrhosis, liver cancer, breast cancer, hemangioma	Zhang, (2015)
Baiying qinghou decoction	*Solanum lyratum* Thunberg, ***S. Nigrum***, *Duchesnea indica* (Andr.) Focke, *Scutellaria barbata* D. Don, *Armeniaca mume* Sieb	Take orally	Decocting with water	Laryngeal cancer	“*Qiu Yuan Ying Fang*”
Kaizhi longgu san	*Os draconis* (FossiliaOssiaMastodi), *Asarum sieboldii* Miq., *Gypsum Fibrosum*, *Ligusticum sinense* Oliv., ***S. Nigrum***, *Angelica dahurica* (Fisch. ex Hoffm.) Benth. et Hook. f., *Gypsum Rubrum*, *S*alt	External use	Making into powder	Bad breath	“*Pu Ji Fang*” (Ming Dynasty, A.D. 1390)
Lingxian longcao decoction	*Clematis chinensis* Osbeck, ***S. Nigrum***, *Prunella vulgaris* Linn., *Smilax glabra* Roxb*.*, *Trichosanthes kirilowii* Maxim., *Clematis terniflora* DC., *Iphigenia indica* Kunth, *Wikstroemia indica* (Linn.) C. A. Mey	Take orally	Decocting with water	Phlegm scrofula, breast mass, wheezing, vomiting	“*Yan Fang Xuan Bian*” (A.D. 1959)

In Libya, *S. nigrum* is often used as folk medicine, and its berries are used as diuretics, antispasmodic, and emetics, and to treat diarrhea, fever, and eye problems, as well as bleeding. In addition, *S. nigrum* leaves are used as a sedative, cholagogic, and anesthetic for the treatment of insomnia, convulsions, and dysentery as well as the external treatment of wounds and itching. In Italy, the aboveground part of *S. nigrum* is used as an antispasmodic, sedative, and analgesic drug. In Yemen, *S. nigrum* is used to dispel phlegm and treat diarrhea and bleeding. In Jordan, *S. nigrum* fruit is used as an antispasmodic drug. Moreover, *S. nigrum* is used as an important plant in traditional Indian medicines for the treatment of dysentery, stomach complaints, and fever (Aburjai et al., 2014).

## Phytochemical Constituents

**FIGURE 2:**
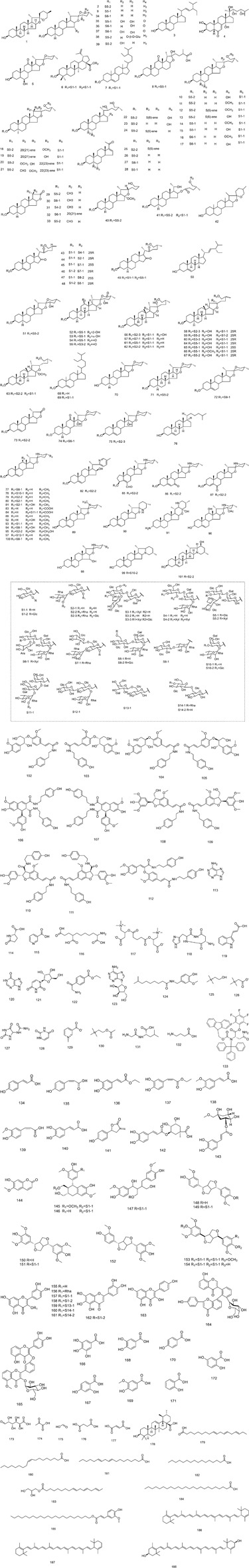
Chemical structures of compounds isolated from *S. nigrum*.

*S. nigrum* is a rich source of natural compounds with varying structural patterns and beneficial properties. To date, approximately 188 phytochemical compounds have been separated and identified from *S. nigrum*, containing steroids, alkaloids, organic acids, flavonoids, phenylpropanoids and their glycosides, as well as other compounds. Steroidal compounds consist of steroidal saponins (**1–76**) and steroidal alkaloids (**77–101**) and are considered to be the main bioactive components of *S. nigrum*, exhibiting various pharmacological activities, such as antitumor, anti-inflammatory, and antiviral activities. The compounds isolated from *S. nigrum* are documented and listed in Table 2, and their chemical structures are drawn and presented in Figure 2. In addition, *S. nigrum* is rich in a large number of polysaccharides, which is a material basis for its various pharmacological activities, such as immunomodulatory and antitumor activities, which are summarized in Table 3.

**TABLE 2:** Chemical components structurally identified from *S. nigrum*.

No	Chemical constituents	Molecular formula	CAS	Extracts	References
Steroidal saponins					
1	Diosgenin	C_27_H_42_O_3_	512-04-9	MeOH	Gao et al. (2021)
					Tsuyoshi et al. (2003)
2	Degalactotigonin	C_50_H_82_O_22_	39941-51-0	EtOH	Wang (2007)
3	Stigmasterol	C_29_H_48_O	83-48-7	EtOH	Zhao (2010)
4	Pterosterone	C_27_H_44_O_7_	18089-44-6	EtOH	Zhao (2010)
5	12-keto-porrigenin	C_27_H_42_O_5_	189014-45-7	EtOH	Zhao (2010)
6	28-*O-β-* d-glucopyranosyl betulinic acid 3*β-O-β*-D glucopyranoside	C_42_H_68_O_13_		EtOH	Yang (2014)
7	*β-*daucosterol	C_35_H_60_O_6_	474-58-8	EtOH	Yang (2014)
8	(25*R*)-5*α*-furost-3*β*, 22*α*-diol-12-one-26-carboxylicacid-3-*O-β*-D-glucopyranosy-(1→4)-[*O*-*β*-d-glucopyranosyl-(1→2)]-*O*- *β*-d-glucopyranosyl-(1→4)-*O*-*β*-d-galactopyranoside	C_47_H_76_O_20_		EtOH	Li (2010b)
9	Tigogenin3-*O-β*-d-glucopyranosyl-(1→2)-[*O*-*β*-d-xyloyranosyl-(1→3)]-*O-β*-d-glucopyranosyl-(1→4)-*O-β*-d-galactopyranoside	C_52_H_86_O_21_		EtOH	Li (2010b)
10	Uttroside A	C_57_H_96_O_28_	82003-86-9	EtOH	He et al. (2015), Zhou (2006)
11	Uttroside B	C_56_H_94_O_28_	88048-09-3	EtOH	He et al. (2015), Zhou (2006)
12	(22*α*, 25*R*)-26-*O*-*(β*-d-glucopyranosyl)-22-methoxy-furost-Δ5-3*β*, 26-diol-3-*O-β*-d-glucopyranosyl	C_57_H_94_O_28_		EtOH	He et al. (2015), Zhou (2006)
	-(1→2)-*O-*[*β*-d-xylopyranosyl-(1→3)]-*O-β*-d-glucopyranosyl-(1→4)-*O-β*-d-galactopyranoside				
13	(22*α*, 25*R*)-26-*O*-(*β*-d-glucopyranosyl)-22-hydroxy-furost-Δ5-3*β*, 26-diol-3-*O-β*-d-glucopyranosyl-(1→2)-*O-*[*β*-d-xylopyranosyl-(1→3)]-*O-β*-d-glucopyranosyl-(1→4)-*O-β*-d-galactopyranoside	C_57_H_92_O_28_		EtOH	He et al. (2015), Zhou (2006)
14	(5*α*, 22*α*, 25*R*)-26-*O*-(*β*-d-glucopyranosyl)-22-methoxy-furostan-3*β*, 26-diol-3-*β*-d-glucopyranosyl-(1→2)-*O*-[*β*-d-glucopyranosyl-(1→3)]- *O-β*-d-glucopyranosyl-(1→4)-*O-β*-d-galactopyranoside	C_58_H_98_O_29_	108886-03-9	EtOH	He et al. (2015), Zhou (2006)
15	(5*α*, 22*α*, 25*R*)-26-*O*-(*β*-d-glucopyranosyl-22-hydroxy-furost-3*β*,26-diol-3-*β*-d-glucopyranosyl-(1→2)-*O*-[*β*-d-glucopyranosyl-(1→3)]-*O-β*-d-glucopyranosyl-(1→4)-*O-β*-d-galactopyranoside	C_57_H_96_O_29_		EtOH	He et al. (2015), Zhou (2006)
16	Solanigroside I	C_62_H_104_O_31_		EtOH	He et al. (2015), Zhou (2006)
17	Solanigroside J	C_61_H_102_O_31_	1354759-80-0	EtOH	He et al. (2015), Zhou (2006)
18	Solanigroside K	C_57_H_94_O_28_		EtOH	He et al. (2015), Zhou (2006)
19	Solanigroside L	C_57_H_92_O_28_		EtOH	He et al. (2015), Zhou (2006)
20	Solanigroside M	C_57_H_92_O_28_		EtOH	He et al. (2015), Zhou (2006)
21	Solanigroside N	C_57_H_94_O_28_		EtOH	He et al. (2015), Zhou (2006)
22	Solanigroside R	C_50_H_84_O_23_		EtOH	He et al. (2015), Zhou (2006), Tai et al. (2018)
23	Solanigroside S	C_50_H_86_O_23_		EtOH	He et al. (2015), Zhou (2006)
24	Solanigroside T	C_45_H_84_O_22_		EtOH	He et al. (2015), Zhou (2006)
25	Hypoglaucin H	C_39_H_60_O_15_	50773-43-8	EtOH	Zhou (2006)
26	5*α*-pregn-16-en-3*β*-ol-20-one-lycotetraoside	C_44_H_70_O_21_		EtOH	He et al. (2015), Zhou (2006)
27	Solanigroside A	C_49_H_78_O_24_	1029362-42-2	EtOH	He et al. (2015), Zhou (2006)
28	Solanigroside B	C_45_H_72_O_22_	1029362-44-4	EtOH	He et al. (2015), Zhou (2006)
29	(5*α*, 20*S*)-3*β*, 16*β*-dihydroxy pregn-22-carboxylic acid (22, 16)-lactone-3-*O-β*-d-glucopyranosyl-(1→2)-*O*-[*β*-D-xylopy ranosyl-(1→3)]-*O-β*-d- glucopyranosyl-(1→4)-*O-β*-d-galactopyranoside	C_45_H_72_O_22_		EtOH	He et al. (2015), Zhou (2006)
30	Solanigroside U	C_50_H_80_O_25_		EtOH	He et al. (2015), Zhou (2006)
31	Solanigroside V	C_50_H_80_O_25_		EtOH	He et al. (2015), Zhou (2006)
32	Solanigroside W	C_45_H_70_O_22_		EtOH	He et al. (2015), Zhou (2006)
33	Solanigroside X	C_45_H_72_O_23_		EtOH	He et al. (2015), Zhou (2006)
34	Nigrumnin I	C_55_H_90_O_25_		EtOH	He et al. (2015), Zhou (2006)
35	Solanigroside C	C_51_H_82_O_26_	905914-27-4	EtOH	He et al. (2015), Zhou (2006)
36	Solanigroside D	C_55_H_88_O_27_	905914-28-5	EtOH	He et al. (2015), Zhou (2006)
37	Solanigroside E	C_55_H_88_O_28_	905914-29-6	EtOH	He et al. (2015), Zhou (2006)
38	Solanigroside F	C_56_H_92_O_28_	905914-30-9	EtOH	He et al. (2015), Zhou (2006)
39	Solanigroside G	C_50_H_82_O_23_	905914-31-0	EtOH	He et al. (2015), Zhou (2006)
40	Solanigroside O	C_51_H_86_O_23_		EtOH	He et al. (2015), Zhou (2006)
41	Nigroside A	C_56_H_94_O_29_	386747-86-0	EtOH	He et al. (2015), Zhou (2006)
42	Tigogenin/(25*R*)-5*α*-spirostan-3*β*-ol	C_27_H_44_O_3_		EtOH	Wu (2011)
43	(25*R*)-26-*O-β*-d-glucopyranosyl-cholest-5(6)-en-3*β*, 26-diol-16,22-dione-3*-O-α*-l-rhamnopyranosyl	C_51_H_86_NO_23_		MeOH	Xiang et al. (2018)
	-(1→2)-[*β*-d-glucopyranosyl-(1→3)]-*β*-d-galactopyranoside				
44	(25*R*)-26-*O-β*-d-glucopyranosyl-cholest-5(6)-en-3*β*, 26-diol-16,22-dione-3-*O-α*-l-rhamnopyranosyl	C_45_H_76_NO_18_		MeOH	Xiang et al. (2018)
	-(1→4)-*β*-d-glucopyranoside				
45	(25*S*)-26-*O*-*β*-d-glucopyranosyl-cholest-5(6)-en-3*β*, 26-diol-16, 22-dione-3-*O-α*-l-rhamnopyranosyl	C_57_H_96_NO_27_		MeOH	Xiang et al. (2018)
	-(1→2)-[*α*-Lrhamnopyranosyl-(1→4)]-[*β*-d-glucopyranosyl-(1→6)]-*β*-d-glucopyranoside				
46	(25*R*)-26-*O-β*-d-glucopyranosyl-(1→2)-*β*-d-glucopyranosyl-cholest-5(6)-en-3*β*, 26-diol-16, 22-dione-3-*O-α*-Lrhamnopyranosyl-(1→2)-[*α*-l-rhamnopyranosyl-(1→4)]-[*β*-d-glucopyranosyl-(1→6)]-*β*-d-glucopyranoside	C_63_H_106_NO_32_		MeOH	Xiang et al. (2018)
47	(25*S*)-26-*O*-*β*-d-glucopyranosyl-cholest-5(6)-en-3*β*, 26-diol-16, 22-dione-3-*O-β*-d-glucopyranosyl	C_57_H_96_NO_28_		MeOH	Xiang et al. (2018)
	-(1→6)-*β*-d-glucopyranosyl-(1→3)-[*α*-l-rhamnopyranosyl-(1→2)]-*β*-d-galactopyranoside				
48	(25*R*)-26-*O-β*-d-glucopyranosyl-(1→2)-*β*-d-glucopyranosyl-cholest-5(6)-en-3*β*, 26-diol-16, 22-dione-3-*O-α*-l-rhamnopyranosyl-(1→2)-[*β*-d-glucopyranosyl-(1→3)]-*β*-d-galactopyranoside	C_57_H_96_NO_28_		MeOH	Xiang et al. (2018)
49	(25*R*)-26-*O-β*-d-glucopyranosyl-cholest-5*α*-3*β*, 26-diol-16, 22-dione-3-*O-β*-d-glucopyranosyl-(1→2)-[*β*-d-glucopyranosyl-(1→3)]-*β*-d-glucopyranosyl-(1→4)-*β*-d-galactopyranoside	C_57_H_98_NO_29_		MeOH	Xiang et al. (2018)
50	Stigmast-5, 22-dien-3β-ol	C_29_H_48_O		EtOH	Sharma et al. (2012)
51	Inunigroside A	C_50_H_82_O_23_	1427934-51-7	MeOH	Ohho et al. (2012)
52	Solanigroside Y1	C_51_H_82_O_26_	2098576-14-6	MeOH	Wang et al. (2017)
53	Solanigroside Y2	C_51_H_82_O_26_	2098576-15-7	MeOH	Wang et al. (2017)
54	Solanigroside Y3	C_51_H_80_O_26_	2098576-16-8	MeOH	Wang et al. (2017)
55	Solanigroside Y4	C_45_H_70_O_21_	2098576-17-9	MeOH	Wang et al. (2017)
56	Solanigroside Y5	C_57_H_94_O_28_	2098576-18-0	MeOH	Wang et al. (2017)
57	Solanigroside Y6	C_57_H_94_O_27_	2098576-19-1	MeOH	Wang et al. (2017)
58	Solanigroside Y7	C_63_H_106_O_34_	2098576-20-4	MeOH	Wang et al. (2017)
59	Solanigroside Y8	C_62_H_104_O_33_	2098576-21-5	MeOH	Wang et al. (2017)
60	Solanigroside Y9	C_62_H_104_O_33_	2098576-22-6	MeOH	Wang et al. (2017)
61	(25*R*)-26-*O-β*-D-glucopyranosylfurost-5(6)-ene-3*β*, 22*α*, 26-triol-3-*O-β*-d-glucopyranosyl-(1→2)-[*β*-d-glucopyranosyl-(1→3)]-*β*-d-glucopyranosyl-(1→4)-*β*-d-galactopyranoside	C_57_H_96_O_30_		MeOH	Wang et al. (2017)
62	(25*R*)-26-*O-β*-Dglucopyranosylfurost-5(6)-ene-3*β*, 22*α*, 26-triol-3-*O-α*-l-rhamnopyranosyl-(1→2)-[*α*-l-rhamnopyranosyl-(1→4)]-*β*-d-glucopyranoside	C_57_H_92_O_25_		MeOH	Wang et al. (2017)
63	(25*R*)-26-*O-β*-D-glucopyranosylfurost-5(6)-ene-16*α*-methoxy-3*β*, 26-diol-3-*O-α*-l-rhamnopyranosyl	C_39_H_60_O_16_		MeOH	Wang et al. (2017)
	-(1→2)-[*α*-l-rhamnopyranosyl-(1→4)]-*β*-d-glucopyranoside				
64	(25*R*)-26-*O-β*-d-glucopyranosyl-5*α*-furost-3*β*, 22*α*, 26-triol-3-*O-β*-d-glucopyranosyl-(1→2)-[*β*-d-glucopyranosyl-(1→3)]-*β*-d-glucopyranosyl-(1→4)-*β*-d-galactopyranoside	C_57_H_94_O_30_		MeOH	Wang et al. (2017)
65	(25*S*)-26-*O-β*-d-glucopyranosyl-5*α*-furost-3*β*, 22*α*, 26-triol-3-*O-β*-d-glucopyranosyl-(1→2)-[*β*-d-glucopyranosyl-(1→3)]-*β*-d-glucopyranosyl-(1→4)-*β*-d-galactopyranoside	C_57_H_94_O_30_		MeOH	Wang et al. (2017)
66	(25*R*)-26-*O-β*-Dglucopyranosyl-22*α*-methoxy-5*α*-furost-3*β*, 26-diol-3-*O-β*-d-glucopyranosyl-(1→2)-[*β*-d-glucopyranosyl-(1→3)]-*β*-d-glucopyranosyl-(1→4)-*β*-d-galactopyranoside	C_57_H_96_O_30_		MeOH	Wang et al. (2017)
67	Uttroside B ((25*R*)-26-*O-β*-d-glucopyranosyl-5*α*-furost-3*β*, 22*α*, 26-triol-3-*O-β*-d-glucopyranosyl	C_56_H_94_O_28_	88048-09-3	MeOH	Wang et al. (2017)
	-(1→2)-[*β*-d-xylopyranosyl-(1→3)]-*β*-d-glucopyranosyl-(1→4)-*β*-d-galactopyranoside)				
68	*β-*sitosterol	C_29_H_50_O	83-46-5	EtOH	Yang (2014)
69	*β*-carotene glycosides	C_35_H_58_O_6_		EtOH	Yang (2014)
70	Tigogenin	C_27_H_44_O_3_	77-60-1	EtOH	He et al. (2015), Zhou (2006)
71	Uttronin A	C_50_H_82_O_22_	39941-51-0	EtOH	He et al. (2015), Zhou (2006)
72	Uttronin B	C_39_H_62_O_12_	84955-03-3	EtOH	He et al. (2015), Zhou (2006)
73	Dumoside	C_40_H_62_O_16_	221526-58-5	EtOH	He et al. (2015), Zhou (2006)
74	Nigrumnin II	C_55_H_88_O_27_		EtOH	He et al. (2015), Zhou (2006)
75	Solanigroside H	C_51_H_82_O_22_	905914-32-1	EtOH	He et al. (2015), Zhou (2006)
76	Cholesterol	C_27_H_46_O	57-88-5	EtOH	Geng et al. (2020)
Alkaloids					
77	*β*1-solasonine	C_39_H_63_NO_11_	73069-18-8	EtOH	He et al. (2015), Zhou (2006)
78	*β*2-solasonine	C_39_H_63_NO_12_	73069-19-9	EtOH	He et al. (2015), Zhou (2006)
79	Solamargine	C_45_H_73_NO_15_	20311-51-7	EtOH	He et al. (2015), Zhou (2006), Wang (2007)
80	*β*2-solamargine	C_39_H_63_NO_11_	32449-98-2	EtOH	He et al. (2015), Zhou (2006) Wang (2007)
81	Solanigroside P	C_39_H_63_NO_12_	1446029-15-7	EtOH	He et al. (2015), Zhou (2006)
82	Solanigroside Q	C_45_H_69_NO_15_		EtOH	He et al. (2015), Zhou (2006), Tai et al. (2018)
83	(3*β*, 12*β*, 22*α*, 25*R*)-3, 12-dihydroxy-spirosol-5-en-27-oic acid	C_27_H_41_NO_5_		EtOH	He et al. (2015), Zhou (2006)
84	Solaoiacid	C_44_H_83_NO_19_		H_2_O	Shi et al. (2019)
85	(25*R*)-22*α*N-4-nor-spirosol-5(6)-en-3*β*-ol-6-al-3-*O*-l-rhamnopyranosyl-(1→2)-[*α*-l-rhamnopyranosyl-(1→4)]-*β*-d-glucopyranoside	C_45_H_72_NO_16_		MeOH	Xiang et al. (2019)
86	(25*R*)-22*α*N-spirosol-5(6)-en-3*β*-ol-7-oxo-3-*O*-l-rhamnopyranosyl-(1→2)-[*α*-l-rhamnopyranosyl-(1→4)]-*β*-d-glucopyranoside	C_45_H_72_NO_16_		MeOH	Xiang et al. (2019)
87	(25*R*)-22*α*N-spirosol-4(5)-en-3*β*-ol-6-oxo-3-*O-α*-Lrhamnopyranosyl-(1→2)-[*α*-l-rhamnopyranosyl	C_45_H_72_NO_16_		MeOH	Xiang et al. (2019)
	-(1→4)]-*β*-d-glucopyranoside				
88	Solasodine	C_27_H_43_NO_2_	126-17-0	EtOH	He et al. (2015), Zhou (2006)
89	N-methylsolasodine	C_28_H_45_NO_2_	7604-92-4	EtOH	He et al. (2015), Zhou (2006)
90	Tomatidenol	C_27_H_43_NO_2_	546-40-7	EtOH	He et al. (2015), Zhou (2006)
91	Solanocapsine	C_27_H_46_N_2_O_2_	639-86-1	EtOH	Liu et al. (2020), Zhou (2006)
92	Solanaviol	C_27_H_43_NO_3_	74131-93-4	EtOH	He et al. (2015), Zhou (2006)
93	Solasodine-3-*O*-*β*-d-glucopyranoside	C_33_H_53_NO_7_		EtOH	Chang et al. (2017)
94	12*β*-hydroxysolasodine *β*-solatrioside	C_45_H_73_NO_17_		EtOH	He et al. (2015), Zhou (2006)
95	12*β*, 27-dihydroxy solasodine *β*-chacotrioside	C_45_H_73_NO_17_		EtOH	He et al. (2015), Zhou (2006)
96	23-*O*-acetyl-12*β*-hydroxysolasodine	C_29_H_45_NO_15_	117803-97-1	EtOH	He et al. (2015), Zhou (2006)
97	(3*β*, 22*α*, 25*R*)-spirosol-5-en-3yl-*O-α*-l-Rhamanopyranosyl-(1-2)-[*O-β*-d-glucopyranosyl(1–3)]-*O-β*-d-galactopyranoside	C_39_H_85_NO_15_	101009-59-0	EtOH	Yang (2014)
98	15*α*-hydroxysolasodine	C_27_H_43_NO_3_	10009-88-8	EtOH	Wu (2011)
99	*α*-Solanine	C_45_H_73_NO_15_	20562-02-1	EtOH	Huang et al. (2020)
100	Solasonine	C_45_H_73_NO_16_	19121-58-5	EtOH	He et al. (2015), Zhou (2006)
101	Leptinine I	C_45_H_73_NO_15_		EtOH	Gao et al. (2021)
102	(7*R*, 8*S*)-1-(4-hydroxy-3-methoxyphenyl)-2-{4-{2-[N-2-(4-hydroxyphenyl)ethyl]Carbamoylehenyl	C_28_H_32_NO_8_		EtOH	Li et al. (2019)
	-2-methoxyphenoxyl}}-1, 3-propanodiolnamed				
103	(7*S*, 8*R*)-1-(4-hydroxy-3-methoxyphenyl)-2-{4-{2-[N-2-(4-hydroxyphenyl)ethyl]Carbamoylehenyl	C_28_H_32_NO_8_		EtOH	Li et al. (2019)
	-2-methoxyphenoxyl}}-1, 3-propanodiolnamed				
104	(7*R*, 8*R*)-1-(4-hydroxy-3-methoxyphenyl)-2-{4-{2-[N-2-(4-hydroxyphenyl)ethyl]Carbamoylehenyl	C_28_H_32_NO_8_		EtOH	Li et al. (2019)
	-2-methoxyphenoxyl}}-1, 3-propanodiolnamed				
105	(7*S*, 8*S*)-1-(4-hydroxy-3-methoxyphenyl)-2-{4-{2-[N-2-(4-hydroxyphenyl)ethyl]Carbamoylehenyl-2-methoxyphenoxyl}}-1, 3-propanodiolnamed	C_28_H_32_NO_8_		EtOH	Li et al. (2019)
106	7′*S*, 8′*R*-7-hydroxy-1-(4-hydroxy-3-methoxyphenyl)-N^2^, N^3^-bis(4-hydroxyphenethyl)-6-methoxy	C_36_H_37_N_2_O_8_		EtOH	Li et al. (2019)
	-1, 2-dihydronaphthalene-2, 3-dicarboxamide				
107	7′*R*, 8′*S*-7-hydroxy-1-(4-hydroxy-3-methoxyphenyl)-N^2^, N^3^-bis(4-hydroxyphenethyl)-6-methoxy	C_36_H_37_N_2_O_8_		EtOH	Li et al. (2019)
	-1, 2-dihydronaphthalene-2, 3-dicarboxamide				
108	7′*R*, 8′*S*-7-(4-hydroxy-3, 5-dimethoxyphenyl)-3′-hydroxymethyl-1′-[N-7″-(4″-hydrxyphenyl)ethyl] carbamoylethenyl-3′-methoxybenzodihydrofuran	C_29_H_31_NO_8_		EtOH	Li et al. (2019)
109	7′*S*, 8′*R*-7-(4-hydroxy-3, 5-dimethoxyphenyl)-3′-hydroxymethyl-1′-[N-7″-(4″-hydrxyphenyl)ethyl] carbamoylethenyl-3′-methoxybenzodihydrofuran	C_29_H_31_NO_8_		EtOH	Li et al. (2019)
110	(7′*R*, 8′*R*)-2-(4-Hydroxy-3-methoxyphenyl)-3-[N-2-(4-hydroxyphenyl)ethyl]carbamoyl-5-[N-2-(4-hydroxyphenyl)ethyl]carbamoylethenyl-7-methoxybenzodihydrofurn	C_36_H_36_N_2_O_8_		EtOH	Li et al. (2019)
111	(7′*S*, 8′*S*)-2-(4-Hydroxy-3-methoxyphenyl)-3-[N-2-(4-hydroxyphenyl)ethyl]carbamoyl-5-[N-2-(4-hydroxyphenyl)ethyl]carbamoylethenyl-7-methoxybenzodihydrofurn	C_36_H_36_N_2_O_8_		EtOH	Li et al. (2019)
112	Cannabisin F	C_36_H_36_N_2_O_8_	163136-19-4	EtOH	Li et al. (2019)
113	Adenine	C_5_H_5_N_5_	73-24-5	MeOH	Gao et al. (2021)
114	Pyroglutamic acid	C_5_H_7_NO_3_	98-79-3	MeOH	Gao et al. (2021)
115	Nicotinic acid	C_6_H_5_NO_2_	59-67-6	MeOH	Gao et al. (2021)
116	9-aminononane-1,3,9-tricarboxylic acid	C_12_H_21_NO_6_		MeOH	Gao et al. (2021)
117	Glutarylcarnitine	C_12_H_21_NO_6_	102636-82-8	MeOH	Gao et al. (2021)
118	(6*S*)-3-((1H-imidazol-4-yl)methyl)-6-amino-1, 4-diazocane-2, 5, 8-trione	C_10_H_13_N_5_O_3_		MeOH	Gao et al. (2021)
119	3-Indoleacrylic acid	C_11_H_9_NO_2_		MeOH	Gao et al. (2021)
120	6-Hydroxypurine	C_5_H_4_N_4_O	68-94-0	MeOH	Gao et al. (2021)
121	Uridine	C_9_H_12_N_2_O_6_	58-96-8	MeOH	Gao et al. (2021)
122	Ethyl 4-glycylbenzoate	C_11_H_13_NO_3_		MeOH	Gao et al. (2021)
123	Adenosine	C_10_H_13_N_5_O_4_	58-61-7	MeOH	Gao et al. (2021), Wang (2007)
124	Dihydrocapsaicin	C_18_H_29_NO_3_	19408-84-5	MeOH	Gao et al. (2021)
125	Choline	C_5_H_14_NO	62-49-7	MeOH	Gao et al. (2021)
126	Betaine	C_5_H_11_NO_2_	107-43-7	MeOH	Gao et al. (2021)
127	Allantoin	C_4_H_6_N_4_O_3_	97-59-6	MeOH	Gao et al. (2021)
128	Uracil	C_4_H_4_N_2_O_2_	66-22-8	MeOH	Gao et al. (2021)
129	Trigonelline	C_7_H_7_NO_2_	535-83-1	MeOH	Gao et al. (2021)
130	(2-acetoxyethyl)trimethylammonium	C_7_H_16_NO_2_^+^		MeOH	Gao et al. (2021)
131	Glycyl-l-leucine	C_8_H_16_N_2_O_3_		MeOH	Gao et al. (2021)
132	GABA	C_4_H_9_NO_2_	56-12-2	MeOH	Gao et al. (2021)
133	FMoc-Asn(Trt)-OPfp	C_44_H_31_F_5_N_2_O_5_		MeOH	Gao et al. (2021)
Phenylpropanoids					
134	*Trans*-4-Hydroxycinnamic acid	C_9_H_8_O_3_	501-98-4	EtOH	Liu et al. (2019b)
135	*Cis*-4-Hydroxycinnamic acid	C_9_H_8_O_3_	4501-31-9	EtOH	Liu Z et al. (2019)
136	Ethyl 3, 4-dihydroxycinnaMate	C_11_H_12_O_4_	102-37-4	EtOH	Liu et al. (2019a)
137	*Cis*-caffeic acid ethyl ester	C_11_H_12_O_4_	74257-25-3	EtOH	Liu et al. (2019b)
138	*Trans* ferulic acid	C_10_H_10_O_4_	537-98-4	EtOH	Liu Z et al. (2019)
139	*Cis*-ferulic acid	C_10_H_10_O_4_	1014-83-1	EtOH	Liu et al. (2019a)
140	Caffeic acid	C_9_H_8_O_4_	331-39-5	MeOH	Gao et al. (2021)
141	4-(4-hydroxyphenyl)-2-methylenebutyrolactone	C_11_H_10_O_3_		EtOH	Liu et al. (2019b)
142	Chlorogenic acid	C_16_H_18_O_9_	327-97-9	MeOH	Gao et al. (2021)
143	3-caffeoylquinic acid methyl ester	C_17_H_20_O_9_	123483-19-2	MeOH	Xiang et al. (2019)
144	Scopoletin	C_10_H_8_O_4_	92-61-5	EtOH	Zhao (2010), Wang et al. (2007), Wang (2007)
145	(−)-5′-methoxyisolariciresinol-3*α*-*O-β*-d-glucopyranoside	C_27_H_36_O_12_		MeOH	Xiang et al. (2019)
146	(+)-isolariciresinol-3*α-O-β*-d-glucopyranoside	C_26_H_34_O_11_		MeOH	Xiang et al. (2019)
147	Cinnacassoside A	C_26_H_36_O_12_	1691248-24-4	MeOH	Xiang et al. (2019)
148	Pinoresinol	C_20_H_22_O_6_	81446-29-9	EtOH	Zhao (2010)
149	Pinoresinol-4-*O-β*-d-glucopyranoside	C_26_H_32_O_11_	69251-96-3	EtOH	Wang et al. (2007), Wang (2007)
150	Syringaresinol	C_22_H_26_O_8_	487-35-4	EtOH	Zhao (2010)
151	Syringaresinol-4-*O-β*-d-glucopyranoside	C_28_H_36_O_13_	137038-13-2	EtOH	Wang et al. (2007), Wang (2007)
152	Medioresinol	C_21_H_24_O_7_	40957-99-1	EtOH	Zhao (2010)
153	Acanthoside D	C_34_H_46_O_18_	573-44-4	MeOH	Xiang et al. (2019)
154	(+)-medioresonol-di-*O-β*-d-glucopyranoside	C_33_H_44_O_17_		MeOH	Xiang et al. (2019)
Flavonoids					
155	Quercetin	C_15_H_10_O_7_	117-39-5	EtOH	Yang (2014)
156	Quercitrin	C_21_H_20_O_11_	522-12-3	EtOH	Yang (2014)
157	Isoquercitrin	C_21_H_20_O_12_	21637-25-2	EtOH	Yang (2014)
158	Quercetin-3-*O-β*-d-glucopyranosyl(1-2)-*β*-d-glucopyranoside	C_27_H_30_O_17_		EtOH	Yang (2014)
159	Quercetin-3-*O-β*-d-galactopyranosyl-(1→6)-*β*-d-glucopyranoside	C_28_H_32_O_17_		MeOH	Xiang et al. (2019)
160	Quercetin-3-gentiobioside	C_27_H_30_O_17_	7431-83-6	EtOH	Wu (2011)
161	Quercetin-3-O-*α*-l-rhaopyranosyl(1→4)-*O-β*-d-glucopyranosyl-(1→6)-*O-β*-d-glucopyranoside	C_28_H_32_O_17_		EtOH	Li (2010b)
162	6-Hydroxyluteolin 7-sophoroside	C_27_H_30_O_17_		MeOH	Gao et al. (2021)
163	Kaempferol	C_15_H_10_O_6_	520-18-3	EtOH	Liu Z et al. (2019)
164	(8-hydroxy-3′-*β*-d-galactosyl-isoflavone)-2′-8″-(4‴-hydroxy-flavone)-biflavone	C_36_H_28_O_11_		EtOAc	Sabudak et al. (2017)
165	2′, 3′, 5-trihydroxy-5″-methoxy-3″-O- *α*-glucosyl-3-4‴-*O*-biflavone	C_36_H_30_O_15_		EtOAc	Sabudak et al. (2017)
Benzoic acids					
166	Gallic acid	C_7_H_6_O_5_	149-91-7	MeOH	Gao et al. (2021)
167	2, 4-Dihydroxybenzoic acid	C_7_H_6_O_4_	89-86-1	MeOH	Gao et al. (2021)
168	Protocatechuic acid	C_7_H_6_O_4_	99-50-3	EtOH	Zhao (2010)
169	Vanillic acid	C_8_H_8_O_4_	121-34-6	EtOH	Zhao (2010)
170	4-Hydroxybenzoic acid	C_7_H_6_O_3_	99-96-7	EtOH	Liu et al. (2019a)
171	Salicylic acid	C_7_H_6_O_3_	69-72-7	EtOH	Liu et al. (2019b)
172	2, 5-Dihydroxybenzoic acid	C_7_H_6_O_4_	490-79-9	MeOH	Gao et al. (2021)
Other compounds					
173	Galacturonic acid	C_6_H_10_O_7_	14982-50-4	MeOH	Gao et al. (2021)
174	Pyruvic acid	C_3_H_4_O_3_	127-17-3	MeOH	Gao et al. (2021)
175	Formic acid	CH_2_O_2_	64-18-6	MeOH	Gao et al. (2021)
176	Succinic acid	C_4_H_6_O_4_	110-15-6	MeOH	Gao et al. (2021)
177	Fumaric acid	C_4_H_4_O_4_	110-17-8	MeOH	Gao et al. (2021)
178	Ursolic acid	C_30_H_48_O_3_	77-52-1	EtOH	Zhao (2010)
179	Linolenic acid	C_18_H_30_O_2_	463-40-1	MeOH	Gao et al. (2021)
180	Oleic acid	C_18_H_34_O_2_	112-80-1	EtOH	Wang (2007)
181	Linoleic acid	C_18_H_32_O_2_	60-33-3	EtOH	Wang (2007)
182	Palmitic acid	C_16_H_32_O_2_	57-10-3	EtOH	Wang (2007)
183	1-monolinolenin	C_21_H_36_O_4_	75685-85-7	EtOH	Zhao (2010)
184	Lignoceric acid	C_24_H_48_O_2_	557-59-5	EtOH	Zhao (2010)
185	(*E*)-docosyl-3-(4-hydroxy-3-methoxyphenyl)acrylate	C_32_H_54_O_4_		EtOH	Zhao (2010)
186	*α*-carotene	C_40_H_56_	432-70-2	EtOH	Wu (2011)
187	*β*-carotene	C_40_H_56_	7235-40-7	EtOH	Wu (2011)
188	Xanthophyll	C_40_H_56_O_2_	127-40-2	EtOH	Wu (2011)

**TABLE 3:** Monosaccharides composition, molecular weight, structures, and bioactivities of polysaccharides purified from *S. nigrum*.

No.	Polysaccharides	Monosaccharide composition	M.W. (Da)	Structures	Bioactivities	References
1	S1	mannose, glucose, galactose, arabinose in a ratio of 1.00: 8.32: 6.72: 2.90	5.44 × 10^4^	ND	Prebiotic effects	Yao et al. (2020)
2	S2	rhamnose, galacturonic acid, glucose, galactose, arabinose in a ratio of 1.00: 0.76: 4.57: 7.25: 4.49	7.22 × 10^4^	ND	Prebiotic effects	Yao et al. (2020)
3	SNL-1	rhamnose, xylose, arabinose, glucose in a ratio of 4.9: 1: 2. 4: 13	5.30 × 10^3^	*α*-glycosidic and *β*- glycosidic linkages	ND	Xiao and Zeng (1998)
4	SNL-2	glucose, arabinose in a ratio of 13.3: 1	1.12 × 10^4^	*α*-glycosidic and *β*- glycosidic linkages	ND	Xiao and Zeng (1998)
5	SNL-3	xylose, mannose, glucose, galactose in a ratio of 75.7: 9.2: 4.9: 10.2	2.37 × 10^4^	1, 3 xyl residue linkage	Immunomodulatory activity	Xiao and Zeng (2000)
6	SNL-4	xylose, mannose galactose in a ratio of 89.4: 1.7: 8.9	4.77 × 10^4^	1, 3 xyl residue linkage	Immunomodulatory activity	Xiao and Zeng (2000)
7	SNL-WP-1	galactose, glucose, mannose, arabinose in a ratio of 1.8: 1: 1.2: 2	1.41 × 10^4^	*β*-glycosidic linkages	Antitumor activity	Li (2010a)
8	SNL-WP-2	galactose, glucose, mannose, xylose, arabinose in a ratio of 5.4:10.4: 1.4: 1: 1.6	9.12 × 10^3^	ND	Antitumor activity	Li (2010a)
9	SNL-AP-1	galactose, glucose, mannose, xylose, arabinose in a ratio of 28: 1: 6: 51: 13	>1.6× 10^6^	*β*-glycosidic linkages	Antitumor activity	Li (2010a)
10	SNL-AP-2	galactose, glucose, mannose, xylose, arabinose in a ratio of 5.8: 1: 1.3: 2.6: 2	>1.6× 10^6^	*β*-glycosidic linkages	Antitumor activity	Li (2010a)
11	SNLBP	xylose, mannose, glucose, galactose in a ratio of 82.2: 7.4: 3.8: 6.6	ND	*β*-glycosidic linkages	Immunomodulatory activity	Liu (2003)
12	SNL-P1a	rhamnose, xylose, arabinose, glucose	ND	*β* (1→3) glycosidic bond	Immunomodulatory and antitumor activity	Li (2009)

### Steroidal Saponins

Steroidal saponins are an important class of secondary metabolites and pharmaceutical resources distributed in higher plants and some marine organisms, showing good pharmacological activities*.* Modern research suggests that steroidal saponins are the major pharmacologically active constituents of *S. nigrum*. Until now, 76 steroidal saponins (**1–76**) have been isolated and identified. Current research on the pharmacological activity of *S. nigrum* is mainly focused on the antitumor and anti-inflammatory activities, and research on the corresponding chemical compositions is mainly focused on the various types of steroidal saponins. In 2006, 22 new steroidal saponins (Solanigroside A-O, Solanigroside R-X) were isolated from the whole plant of *S. nigrum*, and structure-activity analysis of the steroidal saponins in *S. nigrum* showed that the cytotoxic activities of spirostanol saponins and progesterone saponins were stronger than those of furostanol saponins and cholesteric saponins (Zhou, 2006). In 2017, Wang et al. (2017), isolated nine new steroidal saponins from the berries of *S. nigrum* of which Solanigroside Y1 showed significant anti-inflammatory activity. Subsequently, Xiang et al. isolated seven new steroidal saponins (**61–67**) with a new cholestane 16, 22-dione skeleton from immature *S. nigrum* berries, and some of these compounds exhibited moderate anti-inflammatory activity (Xiang et al., 2018). The contents of the steroidal saponins are shown in Table 2, and their structures are presented in Figure 2.

### Alkaloids

Until now, the alkaloids contained in *S. nigrum* reported in the literature are mainly steroidal alkaloids, and most of them are present in the form of glycosides in the fruits, stems, leaves, and roots of the plant. The immature fruit of *S. nigrum* has the highest content of steroidal alkaloids of up to 4.2%, which gradually decreases as the plant grows. This phenomenon may explain the self-protective effect of the plant, as the toxicity of *S. nigrum* alkaloids prevents the young leaves and fruits from being eaten by other animals and promotes the survival of the species. The steroidal alkaloids contained in *S. nigrum* are also the basis of the antitumor activity of *S. nigrum*. Among the steroidal alkaloids contained in *S. nigrum*, solasonine (**100**) and solamargine (**79**) make up to 0.2% and 0.25%, respectively, and the glycoside of solasonine and solamargine formed after alkaline hydrolysis is solasodine (**88**) (He et al., 2015). Solamargine (**79**) is the main component of the total alkaloids of *S. nigrum*, and pharmacological studies have shown that solamargine (**79**) has strong inhibitory activity against liver cancer, cervical cancer, lung cancer, laryngeal cancer, cholangiocarcinoma, esophageal cancer, etc (Wang 2007; Sun et al., 2010; Ding et al., 2012a; Zhang, 2018; Xiang et al., 2019). In addition, *β*_*2*_-solasonine (**78**), solaoiacid (**84**), (25*R*)-22*α*N-4-nor-spirosol-5(6)-en-3*β*-ol-6-al-*3-O*-l-rhamnopyranosyl-(1→2)-[*α*-l-rhamnopyranosyl-(1→4)]-*β*-d-glucopyranoside (**85**), and solasonine (**100**) showed antitumor and anti-inflammatory activities (Xiang et al., 2019; Li et al., 2020; Zhang et al., 2021).

In addition to steroidal alkaloids found in *S. nigrum*, other types of alkaloids have also been identified in *S. nigrum*. Lignanamides are a rare kind of natural product defined as blignans bearing amide groups, displaying diverse biological activities, including neuroprotective, anti-inflammatory, and insecticidal effects. Findings indicated that cannabisin F (**112**) isolated from the above-ground parts of *S. nigrum* has significant neuroprotective activity against MPP^+^-induced SH-SY5Y cell injury models at doses of 12.5, 25, and 50 μM (Li et al., 2019). In 2021, Gao et al. analyzed the chemical constituents of *S. nigrum* using LC-MS and NMR, and identified 89 compounds, mainly including adenine (**113**), nicotinic acid (**115**), 9-aminononane-1, 3, 9-tricarboxylic acid (**116**), adenosine (**123**), allantoin (**127**), and dozens of alkaloids (Gao et al., 2021).

### Phenylpropanoids

Phenylpropanol is a naturally occurring compound composed of a benzene ring connected to three straight-chain carbons (C_6_-C_3_ groups). It has a phenol structure and is a phenolic substance. In the biosynthesis, most of these compounds are formed by a series of reactions such as deamination and hydroxylation of shikimic acid through aromatic amino acids, such as phenylalanine and tyrosine. Until now, 21 phenylpropanoids (**134–154**), including 10 phenylpropionic acid and their esters (134–143), 1 coumarin (144), and 10 lignans (145–154), have been successfully separated and chemically identified by spectroscopic analysis, including 1H-NMR and 13C-NMR, of the whole plants of *S. nigrum*. Scopoletin (**144**) is widely distributed in *S. nigrum*, and current studies have shown that it has various pharmacological activities, such as antitumor, anti-inflammatory, hypoglycemic, hypotensive, and anti-neurodegenerative effects (Chu et al., 2019).

### Flavonoids

Flavonoids are a class of secondary metabolites that account for over half of the plant phenolics, with various pharmacological activities such as antioxidant and anti-inflammatory activities. In 2014, Yang et al. isolated and identified one flavone (**155**) and three flavone glycosides (**156–158**) from the whole plant of *S. nigrum* (Yang, 2014). In 2017, compound (**164**) and (**165**) were isolated and identified from *S. nigrum*, showing that their cholinesterase inhibitory activity was weaker than that of their ethyl acetate extract of *S. nigrum*, presumably due to the synergistic effect between these compounds (Sabudak et al., 2017). The antioxidant activity of *S. nigrum* is closely related to its flavonoid content, and the research on the flavonoids of *S. nigrum* should be increased to provide the basis for the development and utilization of functional foods of *S. nigrum*.

### Benzoic Acids

In addition, seven benzoic acids with phenolic hydroxyl substituents have been identified from *S. nigrum*, including gallic acid (**166**), 2, 4-dihydroxybenzoic acid (**167**), protocatechuic acid (**168**), vanillic acid (**169**), 4-hydroxybenzoic acid (**170**), salicylic acid (**171**), and 2, 5-dihydroxybenzoic acid (**172**) (Wang et al., 2007; Liu et al., 2019b; Gao et al., 2021). Most of these compounds have anti-inflammatory, antioxidant, antibacterial, and antiviral activities, providing broad application prospects and important pharmaceutical intermediates for disease treatment.

### Polysaccharides

Polysaccharides are one of the four substances that form the basis of life activities. More and more research results show that some plant polysaccharides have many special biological activities, such as immune regulative, antifatigue, antioxidative, antiradiative, blood sugar-lowering, antiviral, antitumor, and liver-protective effects (Yang, 2014). At present, 12 kinds of polysaccharides have been successfully isolated and purified from *S. nigrum*, which are reported to have antitumor, immunomodulatory, and liver-protective activities (Liu, 2003; Liu, 2005; Yao et al., 2020). The monosaccharide composition, molecular weight, structural characteristics, and biological activities of the polysaccharides purified from *S. nigrum* are summarized and presented in Table 3.

### Other Compounds

In addition to the above-mentioned compounds, a few compounds (**173–188**) have been identified from *S. nigrum* until now, and the corresponding chemical structures are shown in Figure 2. Compounds (**173–177**) were identified as organic acids. Ursolic acid (**178**) is a famous antitumor triterpene, and compounds (**179–184**) are aliphatic compounds. Compound (**185**) is a ferulic acid ester, which are compounds that are, in addition to *α*-carotene (**186**), *β*-carotene (**187**), and xanthophyll (**188**), essential nutrients for people and of great significance to the health of human eyes and skin (Wang, 2007; Zhao, 2010; Gao et al., 2021).

## Pharmacological Activities

Numerous studies have reported the pharmacological activities of *S. nigrum* in the past decades. Various solvent extracts and isolated bioactive compounds of *S. nigrum* have exhibited many pharmacological properties including antitumor, antioxidant, anti-inflammatory, immunomodulatory, antihypertensive, antimicrobial, and antiviral activities (Yang et al., 2016; Veerapagu et al., 2018; Tian et al., 2019; Guo et al., 2020; Sivaraj et al., 2020). These pharmacological studies have been summarized in Table 4, and the reported effects and mechanisms will be discussed in detail in the following paragraphs. A variety of proprietary Chinese medicines in which *S. nigrum* extract is one of the medicinal ingredients have been widely used in clinical practice. Some of the patents containing *S. nigrum* can be found in Table 5, the pharmacological activities are mostly found in the field of treatment of tumors and skin diseases, mostly in the form of combination with other herbs.

**TABLE 4:** Biological activity of bioactive compounds and extracts of *S. nigrum*.

Biological activities	Extracts/compounds	Types	Testing subjects	Doses/duration	Mechanisms/effects	References
Antitumor activity						
	SNPE	*In vitro*	HepG_2_ cells	0.5, 1.0 and 2.0 mg/ml for 24 h	IC_50_ value was 0.75 mg/ml, arrested the cell cycle at the G2/M phase and CDK1, Bcl-2 and Bid protein expression levels ↓	Wang et al. (2010)
	SNPE	*In vivo*	HepG_2_ tumor-bearing mice	1 or 2 µg/ml for 35 days	Tumor weight and tumor volume ↓	Wang et al. (2011)
	SNWE	*In vitro*	HepG_2_ cells	0.05–2 mg/ml for 24 h	The IC_50_ of SNWE and SNPE was 2.18 and 0.86 mg/ml, respectively, inhibited TPA-induced HepG_2_ migration, TPA-induced PKCα and p38 protein expression levels ↓	Yang et al. (2010)
	SNPE					
	SNWE	*In vitro*	HUVEC and HepG_2_ cells	0.1–2 mg/ml for 24, 48, 72, and 96 h	Suppression of the VEGF-induced activation of AKT and mTOR	Yang et al. (2016)
	SNPE					
	SNWE	*In vivo*	HepG_2_ tumor-bearing mice	0–2% for 35 days	Reduced the volume and weight of the tumors, and CD31 protein expression levels ↓	Yang et al. (2016)
	SNPE					
	SNEE	*In vitro*	A549 cells	100 µg/ml for 16 h	Exhibited specifically stat3-suppressing activity in A549 cells through the decrease of Bcl-xL expression	Park et al. (2014)
	SNTA	*In vitro*	RPMI-8226 cells	12.5, 25, and 50 mg/kg for 14 days	Inhibited I κB-α Phosphorylation and NF-κB/IRF4 signaling pathway to induce apoptosis	Liu et al. (2021)
	SNFME	*In vitro*	C6 cells	0.025–0.4 mg/ml	IC_50_ value was 0.23 mg/ml, attenuated cell cloning, migration and invasion	Li et al. (2021)
	SNWE	*In vitro*	TG-elicited peritoneal macrophages	10–500 mg/ml for 12 h	Decreased NO production and increased the expression of iNOS protein	An et al. (2005)
	SNLP-1	*In vivo*	Lung Cancer Bearing Mice	200 mg/kg/day	Played an antitumor role by enhancing the function of the immune system in the body	Pu (2020)
	SNCE	*In vitro*	786-O cells	40 mg/ml	Inhibited proliferation and promoted apoptosis by inhibiting the activation of PI3K/Akt signaling pathway	Liao et al. (2020)
	2	*In vitro*	Human hepatoma cancer cell line (HepG_2_ cell)	3.125, 6.25, 12.5, 25, 50, and 100 µM	IC_50_ value against HepG2 cell was 0.245 μg/ml	Wang (2007)
	2	*In vitro*	Human tumor cells lines (NCI-H460, SF-268, MCF-7, HepG_2_ Cell)	3.125, 6.25, 12.5, 25, 50, and 100 µM for 48 h	IC_50_ values against four tumor cells were 4.4, 3.1, 1.5, and 0.2 μM, respectively	Zhou (2006)
	26	*In vitro*	Human tumor cells lines (NCI-H460, SF-268, MCF-7, HepG_2_ Cell)	3.125, 6.25, 12.5, 25, 50, and 100 µM for 48 h	IC_50_ values against four tumor cells were 31.8, 34.7, 29.1, and 19.6 μM, respectively	Zhou (2006)
	28	*In vitro*	Human tumor cells lines (NCI-H460, SF-268, MCF-7, HepG_2_ Cell)	3.125, 6.25, 12.5, 25, 50, and 100 µM for 48 h	IC_50_ values against four tumor cells were 52.3, 260.4, 64.7, and 48.6 μM, respectively	Zhou (2006)
	78	*In vitro*	Human tumor cells lines (NCI-H460, SF-268, MCF-7, HepG_2_ Cell)	3.125, 6.25, 12.5, 25, 50, and 100 µM for 48 h	IC_50_ values against four tumor cells were 22.9, 34.2, 42.2, and 19.2 μM, respectively	Zhou (2006)
	79	*In vitro*	Human tumor cells lines (K562, KB, K562/A02, and KB/VCR)	NW	IC_50_ values against four tumor cells were 8.0, 7.8, 5.4, and 7.1 μM, respectively	Zhao (2010)
	79	*In vitro*	HepG_2_	3.125, 6.25, 12.5, 25, 50, and 100 µM	IC_50_ value against HepG2 cell was 19.2 μg/ml	Wang (2007)
	79	*In vitro*	Human tumor cells lines (HL-60, U-937, Jurkat, K562, and HepG_2_)	NW	Exhibited the most potent cytotoxicity to all the cell lines with IC_50_ values of 3.53, 9.31, 2.72, 8.75, 5.36 μM, respectively	Xiang et al. (2019)
	79	*In vitro*	Human tumor cells lines (MDA-MB-231, A549, Hep3B, PC3)	30 and 100 µM	IC_50_ values against 4 tumor cells were 1.86, 2.24, 0.78, 5.13 μM, respectively	Tai et al. (2018)
	79	*In vitro*	Human tumor cells lines (NCI-H460, SF-268, MCF-7, HepG_2_)	3.125, 6.25, 12.5, 25, 50, and 100 µM for 48 h	IC_50_ values against 4 tumor cells were 21.4, 25.1, 15.2, and 7.6 μM, respectively	Zhou (2006)
	79	*In vitro*	K562 cells	5, 7.5 and 10 μM for 0, 2, 4, 6, 8, and 24 h	Induced tumor apoptosis by initiating an early lysosomal destabilization pathway	Sun et al. (2010)
	79	*In vitro*	SMMC-7721 cells	0, 5, 10, 15 μg/ml for 72 h	The IC_50_ values were 9.21 μg/ml	Ding et al. (2012a)
	79	*In vitro*	QBC939 cells	0–10 μΜ	The IC_50_ value was 9.81 μΜ, Inhibited the metastasis and invasion by inhibiting the expression of PI3K/Akt signal pathway	Zhang (2018)
	84	*In vitro*	Lung cancer A549 cells	1–20 μmol/L for 24 h	IC_50_ values against A549 cells were 2.36 μM, respectively	Shi et al. (2019)
	99	*In vitro*	PANC-1 cells	0, 2, 4, 6 μg/ml	N-cadherin, vimentin, MMP2 expression level ↓; E-cadherin expression level ↑	Kong et al. (2020)
	99	*In vitro*	RKO and HCT-116 cells	13–32 µM in RKO cells, 11–28 µM in HCT-116 cells	IC_50_ were 20.84 and 20.32 µM respectively. The expression levels of cyclin D1 and cyclin-dependent kinase 2 in RKO cells↓; production of ROS in RKO cells ↑; inhibited the migration and invasion of HCT-116 cells	Yan et al. (2020)
	99	*In vitro*	HCT-116 cells	4–32 μmol/L for 48 h	Inhibited proliferation and clone, induced apoptosis by activating Caspase-3	Hu et al. (2019)
	99	*In vitro*	SK-OV3 cells	5–15 μmol/L for 24, 48, 72 h	Inhibited proliferation and induced apoptosis by regulating the expression of p-Akt, cleaved Caspase-3 and p53 protein	Zhu et al. (2019)
	99	*In vitro*	RKO cells	NW	Induced apoptosis by activation of Caspase-3, the increase of intracellular ROS level and the inhibition of FAK phosphorylation	Yan and Hu (2019)
	99	*In vitro*	U87 cells	2.5–30 μg/μl	Inhibited proliferation and induced apoptosisby by down-regulating the expression of Ki-67, PCNA and Bcl-2 protein and up-regulating the expression of Bax protein	Zhao et al. (2019)
	99	*In vitro*	SGC-7901 cells	25, 50, 100 μg/ml for 48, 72 h	Inhibited proliferation and promoted apoptosis by up-regulating the expression of mir-140 and down-regulating the expression of MACC1	Huang et al. (2020)
	99	*In vitro*	EC9706, KYSE30 cells	4 μmol/L	Enhanced the drug sensitivity of esophageal cancer cell lines EC9706 and kyse30 to 5-fluorouracil and cisplatin via	Wu (2019)
	99	*In vivo*	ACHN-induced tumor-bearing mice	20 mg/kg for 28 days	Inhibited tumor growth through HIF-1α pathway to affect the expression and activity of key enzymes of glycolysis	Wang et al. (2019)
	100	*In vitro*	THP-1, MV4-11, NB-4, HL-60, HEL cells	NW	IC_50_ were 11.19, 12.50, 15.45, 15.87, 17 mM, promoted apoptosis and caused less cell cycle arrest in the G2/M phase through the activation of the AMPK/FOXO3A Axis	Zhang et al. (2021)
	100	*In vitro*	HepG2 and QGY-7703 cells	0–50 µM for 24, 48, 72 h	The proliferation of hepatoma cells was inhibited by activating mir-375-3p, ccat1, Sp1 and IRF5 protein expression levels ↓	Zhang et al. (2021)
	100	*In vitro*	A549 cells	0, 15, 20, 25 μmol/L for 24, 48, and 72 h	Induced apoptosis by inhibiting the expression of p65 and Bcl-2 protein, enhancing the expression of bik and Bak protein, and activating Caspase-3 pathway	Li et al. (2020)
	100	*In vitro*	Human tumor cells lines (NCI-H460, SF-268, MCF-7, HepG_2_)	3.125, 6.25, 12.5, 25, 50, and 100 µM for 48 h	IC_50_ values against four tumor cells were 97.5, 113.5, 75.7, and 48.9 μM, respectively	Zhou (2006)
	100	*In vitro*	Human tumor cells lines (HL-60, U-937, Jurkat, K562, and HepG_2_)	NW	IC_50_ values against five tumor cells were 33.32, 39.16, 12.85, 26.83, and 17.33 μM, respectively	Xiang et al. (2019)
	180	*In vitro*	HepG_2_	3.125, 6.25, 12.5, 25, 50, and 100 µM	IC_50_ value was 62.3 μg/ml	Wang (2007)
	181	*In vitro*	HepG_2_	3.125, 6.25, 12.5, 25, 50, and 100 µM	IC_50_ value was 57.5 μg/ml	Wang (2007)
Anti-inflammatory activity						
	SNCFE	*In vitro*	Peritoneal macrophages	0–200 μg/ml for 4 and 24 h	NO, TNF-*α* and IL-6 levels ↓; p38, JNK and ERK1/2 expression levels ↓	Kang et al. (2011)
	SNFEE	*In vivo*	Acute ear edema mouse model	0.125, 0.250, 0.500, and 1.000 mg/ml	The cell viability below 0.5 mg/ml was about 90%, alleviating edema and decreased thickness of ear tissue	Yeom et al. (2019)
	SNEE	*In vivo*	Acute and sub-acute rat model	100 and 200 mg/kg	The pathological changes of granuloma, kidney, liver and stomach were lighter than those in the model group	Aryaa and Viswanathswamy (2017)
	SNWE	*In vitro*	Patients with thoracic malignant tumor after radiotherapy	NW	PDGF, TGF-β1, IL-6,TNF-α expression level ↑	Che (2018)
	SNFPEFE	*In vitro*	Hyaluronidase, lipoxygenase	100–1,000 µg/ml	The IC_50_ values of hyaluronidase and lipoxygenase were 810.67 and 781.28 µg/ml	Guo et al. (2020)
	43	*In vitro*	LPS-induced RAW 264.7 cells	2.5, 5, 10, 20, 40, and 50 μM	NO inhibition (IC_50_ = 40.11 μM)	Xiang et al. (2018)
	44	*In vitro*	LPS-induced RAW 264.7 cells	2.5, 5, 10, 20, 40, and 50 μM	NO inhibition (IC_50_ = 72.39 μM)	Xiang et al. (2018)
	45	*In vitro*	LPS-induced RAW 264.7 cells	2.5, 5, 10, 20, 40, and 50 μM	NO inhibition (IC_50_ = 33.00 μM)	Xiang et al. (2018)
	46	*In vitro*	LPS-induced RAW 264.7 cells	2.5, 5, 10, 20, 40, and 50 μM	NO inhibition (IC_50_ = 48.75 μM)	Xiang et al. (2018)
	47	*In vitro*	LPS-induced RAW 264.7 cells	2.5, 5, 10, 20, 40, and 50 μM	NO inhibition (IC_50_ = 50.77 μM)	Xiang et al. (2018)
	48	*In vitro*	LPS-induced RAW 264.7 cells	2.5, 5, 10, 20, 40, and 50 μM	NO inhibition (IC_50_ = 63.66 μM)	Xiang et al. (2018)
	49	*In vitro*	LPS-induced RAW 264.7 cells	2.5, 5, 10, 20, 40, and 50 μM	NO inhibition (IC_50_ = 11.33 μM)	Xiang et al. (2018)
	52	*In vitro*	LPS-induced RAW 264.7 cells	12.5 and 25.0 μM for 24 h	NO inhibition (IC_50_ = 9.7 μM)	Wang et al. (2017)
	53	*In vitro*	LPS-induced RAW 264.7 cells	12.5 and 25.0 μM for 24 h	NO inhibition (IC_50_ = 17.8 μM)	Wang et al. (2017)
	54	*In vitro*	LPS-induced RAW 264.7 cells	12.5 and 25.0 μM for 24 h	NO inhibition (IC_50_ = 14.0 μM)	Wang et al. (2017)
	56	*In vitro*	LPS-induced RAW 264.7 cells	12.5 and 25.0 μM for 24 h	NO inhibition (IC_50_ = 38.3 μM)	Wang et al. (2017)
	57	*In vitro*	LPS-induced RAW 264.7 cells	12.5 and 25.0 μM for 24 h	NO inhibition (IC_50_ = 41.0 μM)	Wang et al. (2017)
	59	*In vitro*	LPS-induced RAW 264.7 cells	12.5 and 25.0 μM for 24 h	NO inhibition (IC_50_ = 48.5 μM)	Wang et al. (2017)
	60	*In vitro*	LPS-induced RAW 264.7 cells	12.5 and 25.0 μM for 24 h	NO inhibition (IC_50_ = 44.0 μM)	Wang et al. (2017)
	63	*In vitro*	LPS-induced RAW 264.7 cells	12.5 and 25.0 μM for 24 h	NO inhibition (IC_50_ = 22.1 μM)	Wang et al. (2017)
	85	*In vitro*	LPS-induced RAW 264.7 cells	NW	NO inhibition (IC_50_ = 23.42 μM)	Xiang et al. (2019)
	164	*In vitro*	BChE assay	NW	moderate BChE inhibitory activity (IC_50_ = 195.2 µg/ml)	Sabudak et al. (2017)
	165	*In vitro*	BChE assay	NW	Moderate BChE inhibitory activity (IC_50_ = 299.1 µg/ml)	Sabudak et al. (2017)
Antioxidant activity						
	SNFME	*In vitro*	DPPH and hydrogen peroxide radicals	25, 50,100, 150, and 200 μg/ml	The IC_50_ value of 70.73 μg/ml for DPPH radical scavenging and IC_50_ 59.72 μg/ml for hydrogen peroxide scavenging activity	Veerapagu et al. (2018)
	SNFEE	*In vitro*	DPPH and hydroxyl radical	0–2.4 mg/ml	The scavenging rate on DPPH, hydroxyl radical scavenging assay were 68.45% and 49.12%, respectively	Teng et al. (2014)
	SNFP	*In vitro*	DPPH and hydroxyl radicals	0–1.2 mg/ml	The IC_50_ values were 65.43 μg/ml and 0.33 mg/ml for DPPH, hydroxyl radical scavenging assay	Chen et al. (2020)
	SNFEAE	*In vitro*	FRAP and DPPH· scavenging assays	100–2,500 μg/ml for FRAP 50–1,000 µg/ml for DPPH	The IC_50_ values were 119.43 µg/ml and 2.674 µg/ml, FeSO4/L for DPPH and FRAP scavenging activity	Guo et al. (2020)
	SNFEE	*In vitro*	DPPH and ABTS radical	0–120 μg/ml	Showed moderate free radical scavenging activity against DPPH and ABTS^+^ free radical with the IC_50_ were 81.02 and 35.56 μg/ml, respectively	Sivaraj et al. (2020)
Immunoregulatory activity						
	SNLWP-1 SNLAP-1 SNLAP-2	*In vivo*	H22-bearing mice	50, 100, and 200 mg/kg for 10 days	IL-2, IFN-c levels ↑; IL-10 levels↓	Ding et al. (2012b)
	SNCP	*In vivo*	Male BALB/C mice	200, 400, 800 mg/kg for 28 days	B.T, NK cell activity ↑	Tian et al. (2019)
	SNLP-1	*In vivo*	Lung Cancer Bearing Mice	200 mg/kg/day	CD_4_+/CD_8_+of T lymphocytes levels ↑; Th1 cytokines levels ↑	Pu (2020)
Hepatoprotective activity						
	SNWE	*in vivo*	CCl_4_-induced chronic hepatotoxicity in rats	0.2, 0.5, and 1.0 g/kg for 6 weeks	GOT, GPT, ALP, total bilirubin, superoxide , hydroxyl radical levels↓; GSH, SOD, GST Al, GST Mu levels ↑	Lin et al. (2008)
	SNFBFE	*in vivo*	D-GalN-induced hepatic fibrosis rats	16 and 25 mg/kg for 10 days	ALT, AST, ALP enzymes, GSH, SOD, and CAT levels↓	Chester et al. (2019)
	SNWSP	*in vivo*	CCl_4_-induced acute injury in rats	100, 200, 400 mg/kg for 7 days	ALT, AST, ALP, MDA levels↓; SOD, GSH-Px, CAT levels ↑	Yang et al. (2014)
	SNWE	*in vivo*	Ethanol-induced liver injury in rats	100, 150, 200 mg/kg for 7 days	ALT, AST, GSTA1, MDA levels↓; SOD, GSH, GSH-Px ↑	Han (2014)
Antibacterial activity						
	SNFEE	*in vitro*	*Aspergillus’s Niger, Fusarium oxysprum*	250–1,000 µg/ml for 24 h	Highest antifungal zone was 32.42 and 28.16 mm against *Aspergillus’s Niger* and *Fusarium oxysprum*	Mazher et al. (2017)
	SNFEE	*in vitro*	*Escherichia coli*	250–625 µg/ml	The maximum zone of inhibition was 25 mm for *Escherichia coli* at 625 µg/ml concentration	Sivaraj et al. (2020)
	SNEE	*in vitro*	*Staphylococcus aureus*	12.5–200 mg/ml	The maximum zones of inhibition were 16.88, 11.33, and 19.25 mm for *Staphylococcus aureus*, *Escherichia coli*, *Aeromona sobria* at 200 mg/ml concentration	Ge (2019)
			*Escherichia coli*			
			*Aeromona sobria*			
	SNEE	*in vitro*	*Alternaria solani*	NW	The EC_50_ values of *Rhizoctonia solani* and *Fusarium oxysporun* were 1,629 and 1,262 ppm	Cai (2003)
			*Cladosporium cucumerinum*			
			*Fusarium oxysporun*			
			*Rhizoctonia solani*			
	93	*in vitro*	*Candida albicans*	0, 8, 16, 32, and 64 mg/L for 12, 24, 36, 48 h	Inhibited the activity of *Candida albicans* via regulating Ras-cAMP-PKA signaling pathway and reducing the intracellular cAMP content	Li et al. (2015)
	93	*in vitro*	*Candida albicans*	32, 64 µg/ml	Alkalizing the intracellular vacuole of *Candida albicans* and causing hyper-permeability of the vacuole membrane	Chang et al. (2017)
Insecticidal activity						
	SNLME	*in vitro*	2nd instar larvae of CPB	5, 10,15, 20, 25, 30, 35, 40, and 45 mg/ml	Caused 50% mortality for 2nd instar CPB larvae at concentration of 5 ppm and foliar consumption was decreased by 74%	Ben-Abdallah et al. (2019)
	SNLCME	*in vitro*	Cx. vishnui group and An. subpictus	25, 45, 60 mg/L for 24, 48, and 72 h	Showed 100 percent larval mortality against early 3rd instar of An. subpictus at 60 mg/L	Rawani et al. (2017)
	SNFMWE	*in vitro*	*Galba truncatula*	NW	The hydro-methanol LC_50_ = 3.96 mg/L, LC_90_ = 7.49 mg/L	Hammami et al. (2011)
	SNLEAE	*in vitro*	*Culex quinquefasciatus*	10–50 ppm for 24–72 h	LC_50_ values of ethyl acetate extracts were 17.04 ppm	Rawani et al. (2010)
	SNLEE	*in vitro*	Green Peach Aphid Myzus persicae Sulzer	4.24 mg/ml for 24, 48, and 72 h	Caused 28.54%, 56.8%, and 57.42% mortality rates after 24, 48, and 72 h exposure	Madanat et al. (2016)
	SNLME	*in vitro*	*Culex quinquefasciatus*	6.25–1,000 ppm	Methanol leaves extract causing 90% mortality rate	Rahuman et al. (2009)
Neuroprotective activity						
	SNL	*in vivo*	SCOP-induced cognitive impairment rats	5% and 10% leaf inclusions	ChEs levels↑; restored the impaired memory function	Ogunsuyi et al. (2018)
	SNL	*in vivo*	AlCl_3_-induced neurodegeneration in *Drosophila melanogaster*	0.1% and 1.0% pulverized vegetable for 7 days	GST, MAO, ChE, ROS, TBARS levels ↓; Athletic, memory ability ↑	Ogunsuyi et al. (2020)
	SNL	*in vivo*	AlCl_3_-induced neurodegeneration in *Drosophila melanogaster*	0.1% and 1.0% pulverized vegetable for 7 days	ROS, GST, Hsp70, Jafrac1, reaper and NF-kҝB/Relish ↓; cnc/Nrf2 and FOXO ↑	Ogunsuyi et al. (2021)
	112	*In vitro*	MPP+-induced SH-SY5Y cells	12.5, 25, and 50 μM for 1 h	Induced protective autophagy to protect SH-SY5Y cells from MPP+-induced apoptosis, the cell viability of which improved by 12% at 25 μM	Li et al. (2019)
Gastroprotective activity						
	SNEE	*in vivo*	Ethanol-induced gastric ulcer mice	5–500 mg/kg	At dose of 500 mg/kg, the extract was as effective as lansoprazole in reducing all parameters of peptic ulcer in both models	El-Meligy et al. (2015)
	SNFME	*in vivo*	Gastric ulcer rats	200 and 400 mg/kg	Gastric secretory volume, acidity, pepsin secretion ↓	Jainu and Devi (2006)
Hypoglycemic activity						
	SNFWE	*in vivo*	Streptozotocin-induced Diabetic rats	1 g/L for 8 weeks	Ca/Mg ratio, plasma glucose, HDL, LDL, VLDL, cholesterol, triglyceride ↓	Sohrabipour et al. (2013)
Antimalarial activity						
	79	*in vivo*	Plasmodium yoelii-infected mice	7.5 mg/kg for 4 days	At a dose of 7.50 mg/kg, the parasitemia suppressions of solamargine were 64.89%, respectively	Chen et al. (2010)
	100	*in vivo*	Plasmodium yoelii-infected mice	7.5 mg/kg for 4 days	At a dose of 7.50 mg/kg, the parasitemia suppressions of solasonine were 57.47%, respectively	Chen et al. (2010)
CNS-depressant activity						
	SNFEE	*in vivo*	Wistar rats and CD1 mice	51, 127.5, and 255 mg/kg	Exploratory and aggressive behavior↓; locomotor activity↓; pentobarbital-induced sleeping time ↑	Perez et al. (1998)
Hypolipidemic activity						
	SNWE	*in vitro*	3T3L1 cells model	0.3, 0.4, 0.5 mg/ml	PPAR*α*, CPT-1 ↑; FaS, HMG-CoR↓; amount and lipid content of adipocytes ↓; inhibiting lipogenesis	Peng et al. (2020)
	SNSEE	*in vivo*	Triton-induced hyperlipidemic rats	200 and 400 mg/kg	Total cholesterol, triglycerides, LDL cholesterol ↓; HDL cholesterol ↑	Sohrabipour et al. (2013)

Note: NM, not mentioned; SNWE, water extracts of S. nigrum; SNPE, polyphenol extracts of S. nigrum; SNEE, ethanol extracts of S. nigrum; SNTA, total alkaloids of S. nigrum; SNFME, Methanol extracts of S. nigrum fruits; SNCE, chloroform Extracts of S. nigrum; SNFEE, ethanol extracts of S. nigrum fruits; SNFP, Polysaccharide from S. nigrum fruit; SNFEAE, Ethyl acetate extracts of S. nigrum fruit; SNCFE, chloroform Fraction extracts of S. nigrum; SNFPEFE, Petroleum ether fraction extracts of S. nigrum fruit; SNCP, Crude Polysaccharides from S. nigrum; SNFBFE, n-butanol fraction extracts of S. nigrum fruit; SNWSP, water-soluble polysaccharides from S. nigrum; SNLME, methanol extracts of S. nigrum leaves; SNLCME, chloroform: methanol (1:1 v/v) extracts of S. nigrum leaves; SNFMWE, methanol-water (8:2 v/v) extracts of S. nigrum fruit; SNLEAE,Ethyl acetate extracts of S. nigrum leaves; SNLEE, ethanol extracts of S. nigrum leaves; SNFWE, water extracts of S. nigrum fruits; SNL, S. nigrum leaves; SNSEE, ethanol extracts of S. nigrum seeds.

**TABLE 5:** Patents list of products containing *S. nigrum* and their claimed pharmacological properties.

Application	Main composition	Pharmacological properties	Publish number
Herbal preparation	*Fritillaria thunbergii* Miq., *Hedyotis diffusa* Willd., ***S. nigrum***, *Paris polyphylla* Smith, *Scutellaria barbata* D. Don, *Adenophora stricta* Miq., *Prunella vulgaris* Linn., etc.	Treating pulmonary fibrosis	CN111991507A
Herbal preparation	*Coix lacryma-jobi* Linn., *Amygdalus persica* Linn., *Carthamus tinctorius* Linn., *Scutellaria baicalensis* Georgi, *Sophora flavescens* Alt., *Rehmannia glutinosa* Libosch., ***S. nigrum***, etc.	Treating lung and colon cancer	CN113398215A
Bacteriostatic agent	***S. nigrum***, *Artemisia argyi* Levl. et Van., *Syzygium aromaticum* (L. ) Merr. Et Perry, *Cynanchum paniculatum* (Bunge) Kitagawa, Carbomer, Salicylic acid, etc.	Treating skin diseases	CN113368193A
Herbal preparation	*Cordyceps Sinensis* (Berk.) Sacc., ***S. nigrum*** extra*ct, Taxus chinensis* (Pilger) Rehd. extract	Treating lung cancer	CN113332358A
Herbal preparation	***S. Nigrum***, *Pteris multifida* Poir., *Lithospermum erythrorhizon* Sieb. et Zucc., *Angelica dahurica* (Fisch. ex Hoffm.) Benth. et Hook. f., *Dioscorea polystachya* Turczaninow	Treating psoriasis	CN110368445A
Herbal preparation	***S. Nigrum***, *Astragalus membranaceus* (Fisch. )Bge., *Panax ginseng* C. A. Meyer, *Atractylodes macrocephala* Koidz., *Hedyotis diffusa* Willd., *Curcuma zedoaria* (Christm.) Rosc., etc	Treating stomach cancer	CN112717097A
Herbal preparation	***S. Nigrum***, *Cinnamomum cassia* Presl, *Dioscorea polystachya* Turczaninow, *Asarum sieboldii* Miq., *Scrophularia ningpoensis* Hemsl., *Cornus officinalis* Sieb. et Zucc., etc	Treating glaucoma	CN108210683A
Herbal preparation	***S. Nigrum***, *Hedyotis diffusa* Willd., *Verbena officinalis* Linn., *Lithospermum erythrorhizon* Sieb. et Zucc*.*, *Reynoutria japonica* Houtt., *Angelica dahurica* (Fisch. ex Hoffm.) Benth. et Hook. f., etc	Treating vaginitis and cervicitis	CN111991481A
Herbal preparation	***S. Nigrum***, *Bidens pilosa* Linn*.*, *Hedyotis diffusa* Willd., *Verbena officinalis* Linn., *Taraxacum mongolicum* Hand.-Mazz., *Plantago asiatica* L., etc	Treating cholecystitis	CN111529631B
Herbal preparation	***S. Nigrum***, *Gentiana scabra* Bunge, *Gardenia jasminoides* Ellis, *Paeonia suffruticosa* Andr., *Angelica sinensis* (Oliv.) Diels, *Pinellia ternata* (Thunb.) Breit., etc	Treating leukemia	CN106822558B

Abbreviation: A549, human alveolar basal epithelial cells; ABTS, 2, 2′-azino-bis-(3-ethylbenzenthiazoline-6-sulphonic) acids; AChE, acetylcholinesterase; AFP, Alpha-FetoProtein; AKT, proteinkinase B; ALP, alkaline phosphatase; AMPK, 5-AMP activated protein kinase; AP-1, activator protein-1; Bax, bcl-associated X protein; Bcl-2, B-cell CLL/lymphoma 2; Caspase 3, cysteinyl aspartate-specific proteinase-3; Caspase 7, cysteinyl aspartate-specific proteinase-7; CAT, catalase; CDC25, recombinant cell division cycle protein 25; CDK1, recombinant cyclin dependent kinase 1; c-JUN, c-Junamino-terminalkinase; COX-2, cyclooxygenase-2; DLD-1, human colorectal adenocarcinoma epithelial cells; DPPH, 1,1-Diphenyl-2-picrylhydrazyl free radical; GOT, glutamic-oxal(o)acetic transaminase; GPT, glutamic pyruvate transaminase; GPx, glutathione peroxidase; GSH, l-glutathione; GST-α, glutathione S-transferase-α; GST-μ, glutathione S-transferase-μ; H22, mouse H22 hepatocellular carcinoma cells; HAase, Human Hyaluronidase; HCT-116, human colorectal adenocarcinoma cells; HDL, high-density lipoprotein; HEL, human erythorleukemia cell line; HepG2, liver hepatocellular cells; HL-60, Leukemia Myeloidcells; HT-29, human conlon carcinoma cells; IC50, half maximal inhibitory concentration; IFN-γ, Interferon-gamma; IL-17, interleukin-17; IL-1β, interleukin-1β; IL-2, interleukin-2; IL-6, interleukin- 6; iNOS, inducible nitric oxide synthase; c-JNK, Jun N-terminal kinase; LC3, microtubule associated proteins 1A/1B light chain 3; LDH, lactate dehydrogenase; LDL, low-density lipoprotein; LT, leukotriene; MACC1, human metastasis associated in colon cancer 1; MCF-7, human breast adenocarcinoma cell line; MDA, malondialdehyde; MIC, minimum inhibitory concentration; mTOR, molecular target of rapamycin; MV4-11, Human acute monocytic Leukemia cells; MyD88, myeloid differentiation primary response protein; NB4, acute promyelocyte cells; NF-κB, nuclear factor kappa-B; NO, nitric oxide; Nrf2, Nuclear Factor erythroid 2-Related Factor 2; PDTC, pyrrolidine dithiocarbamate; PG, prostaglandin; PKCα, recombinant protein kinase C Alpha; ROS, reactive oxygen species; SGC-7901, human gastric cancer cells; SMMC-721, human hepatocellular carcinoma cells; SOD, superoxide dismutase; TG, thioglycollate; THP-1, human monocytic leukemia cells; TLR4, toll like receptor 4; TNF, tumor necrosing factor; TNF-α, tumor necrosis factor -α; TRAF-6, TNF receptor associated factor 6; VEGF, vascular endothlial growth factor; XTT, 2, 3- bis(2-methoxy-4-nitro-5-sulfophenyl)-5-[(phenylamino)carbonyl]-2H-tetrazolium hydroxide; γ-GT, γ-glutamy transpeptidase.

### Antitumor Activity

**FIGURE 3:**
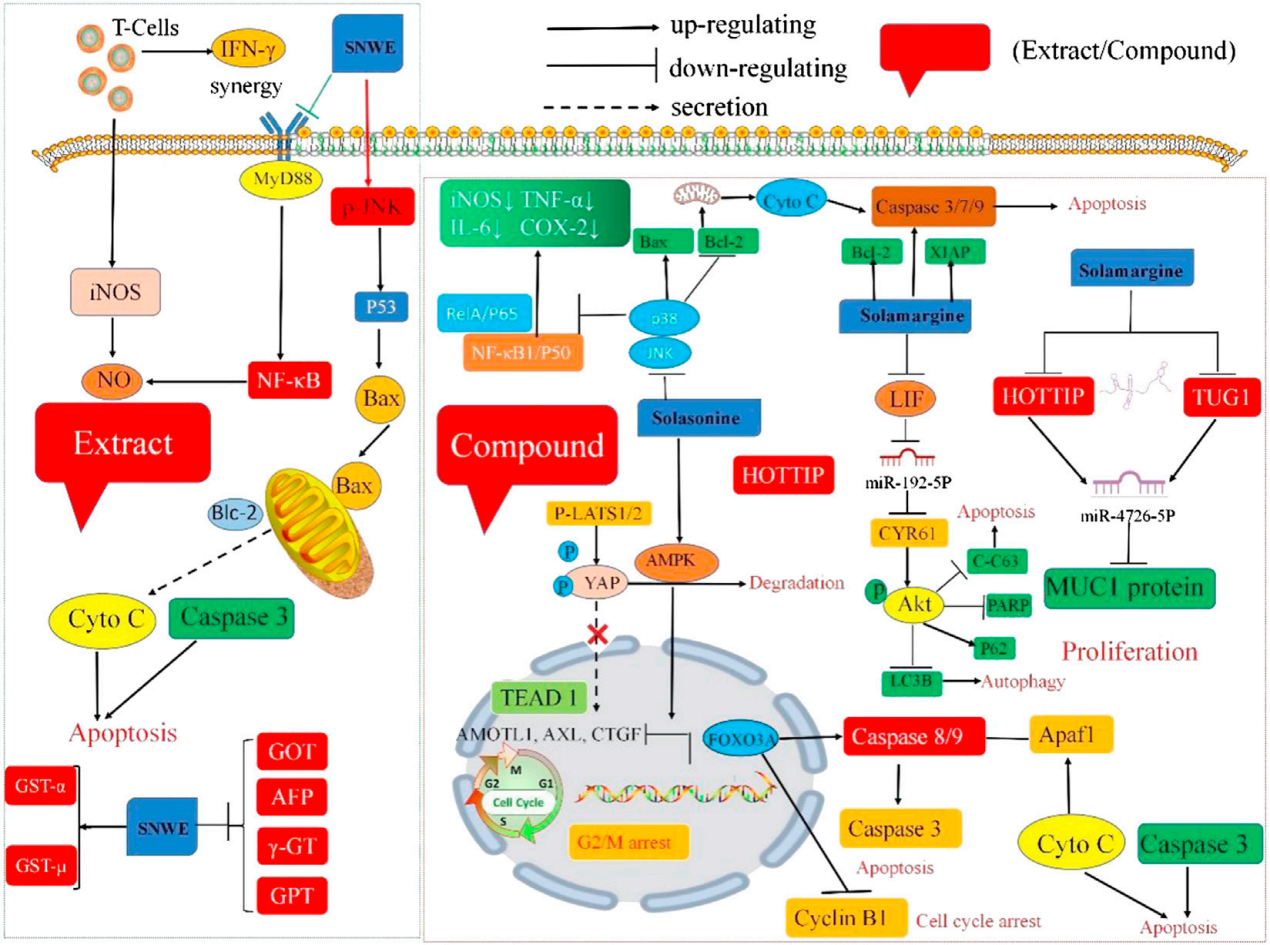
Schematic representation of the molecular mechanism of anti-tumor activities of crude extracts or isolated compounds from *S. nigrum*. (SNWE, water extracts of *S. nigrum*).

Crude extracts and isolated compounds of *S. nigrum* have exhibited significant antitumor potential *in vitro* and *in vivo*. The underlying mechanism of the antitumor activity of the crude extracts or bioactive substances of *S. nigrum* is presented in Table 4 and Figure 3.

### Crude Extract

*In vitro* studies showed that different solvent extracts of *S. nigrum* significantly inhibited the growth of various cancer cell lines, such as human breast cancer cell line MCF-7, renal cell carcinoma cell line 786-O, esophageal cancer cell line ECA-109, human liver cell lines SMMC-721 and HepG_2_, gastric cancer cell line MGC-803, human colorectal carcinoma cell lines HT-29, HCT-116, and DLD-1, and human lung cancer cell line A549 (He et al., 2015). Specifically, the treatment with the water extract of *S. nigrum* (SNWE) induced apoptosis in HepG_2_ cells by increasing the mitochondrial release of cytochrome C, and activating Caspase-3, and inducing autophagy through implicating the levels of LC3, Bcl-2, and Akt (Lin et al., 2007). Nitric oxide (NO) is an antitumor molecule produced by activated macrophages. Harvesting thioglycollate (TG)-elicited peritoneal macrophages from mice followed by incubation with different concentrations of SNWE (10–500 mg/ml) alone or with recombinant interferon-*γ* (rIFN-*γ*) (20 U/ml) for 6 h showed that the extract dose-dependently induced NO production and iNOS expression, which was highly strengthened in combination with rIFN-*γ*. Further mechanism research demonstrated that pyrrolidine dithiocarbamate (PDTC), an NF-κB inhibitor, inhibited the synergistic effect of *S. nigrum* and rIFN-*γ* on the NO production and iNOS expression. These results suggested that *S. nigrum* increased the NO production through NF-κB activation (An et al., 2005). Furthermore, 1% and 2% SNWE prominently reduced hepatic carcinogenesis to 40% and 20% and significantly increased the survival rate to 90% and 100%, respectively, in AAF/NaNO_2_-induced hepatoma rats (Hsu et al., 2009). SNWE also caused 43% cytotoxicity, inhibited migration, and suppressed the activities of hexokinase and pyruvate on the of human breast cancer cell line (MCF-7) by about 30% and 40% at a concentration of 10 g/L, respectively (Ling et al., 2019).

Wang et al. reported that the IC_50_ value of the polyphenolic extract of *S. nigrum* (SNPE) was 0.75 mg/ml and the cell viabilities of HepG_2_ cells were 85%, 27%, and 6% at concentrations of 0.5, 1.0, and 2.0 mg/ml, respectively. Furthermore, SNPE arrested the cell cycle at the G2/M phase (21.13%, 24.53%, and 31.62% at 0.5, 1.0, and 2.0 mg/ml, respectively) by regulating the activity of the CDC25 family and CDK1 and reactivated apoptosis via decreasing protein expression of Bcl-2 and Bid. Moreover, SNPE decreased the tumor weight and tumor volume after feeding HepG2 tumor-bearing mice daily with 5 g basal diet containing 1 or 2 µg/ml (w/v) SNPE for 35 days (Wang et al., 2010). Yang et al. (2010) also revealed that SNPE significantly reduced the viability of HepG_2_ cells (IC_50_ = 0.86 mg/ml). The mechanism of action study showed that SNPE inhibited TPA-induced HepG2 migration and invasion via blocking the expression of PKCα and attenuated p38 and p38/ERK activation (Yang et al., 2010). Moreover, SNPE inhibited the viability of HepG_2_ cells through the suppression of the VEGF-induced activation of AKT and mTOR *in vitro* and reduced the volume and weight of the tumors in the HepG_2_ tumor-bearing mouse model (Yang et al., 2016).

According to relevant literature reports, different solvent extracts (water, ethanol, chloroform, and n-Butanol) extracts of *S. nigrum* show strong broad-spectrum antitumor activity. It has been demonstrated that the ethanol extract of *S. nigrum* fruit (SNCE) could arrest the cell cycle in the S phase and continue to the G2/M phase, inhibiting MCF-7 proliferation (IC_50_ = 40.77 μg/ml) and inducing apoptosis 43.31% (Churiyah et al., 2020). The n-Butanol extract of *S. nigrum* inhibited the growth of human colorectal cancer SW480 cells in a dose-dependent manner via blocking cells in the G2/M phase and increasing the expression of Caspase-3 (Ye and Gao, 2019). These studies indicated that different solvent extracts of *S. nigrum* could be promising candidates for the treatment of cancer.

### Isolated Compounds

Furthermore, compounds isolated from *S. nigrum* also displayed multiple antitumor effects. *α*-solanine (**99**) is a chemical component that widely exists in potatoes, tomatoes, eggplants and other Solanaceae plants. Pharmacological studies have shown that it has antitumor and insecticidal activities, but it also showed toxic effects on humans in overdose. Treatment of the human gastric cancer cell line (SGC-7901) with different concentrations of α-solanine (25, 50, 100 μg/ml) for 24 or 48 h inhibited proliferation and promoted apoptosis of cells by upregulating the expression of miR-140 and downregulating the expression of MACC1 (Huang et al., 2020). Furthermore, α-solanine exhibited antitumor properties by regulating the expression of p-Akt, cleaving Caspase-3 and p53 protein, increasing the intracellular ROS level, and inhibiting FAK phosphorylation and the expression of miR-138 and the survivin protein (Wu, 2011; Zhu et al., 2019). The IC_50_ values of solasonine (**100**) against five tumor cells (THP-1, MV4-11, HL-60, NB4, HEL) were 11.19, 12.50, 15.87, 15.45, and 17.00 mM, respectively. Moreover, solasonine promoted apoptosis and caused less cell cycle arrest in the G2/M phase through the activation of the AMPK/FOXO3A Axis. In the THP-1-induced xenograft model, all the mice were intraperitoneally injected with 4, 8, and 16 mg/kg solasonine once a day for 14 days, and the pharmaceutical and immunoblotting results showed that solasonine inhibited tumor growth with increasing solasonine concentration and induced the nuclear translocation of FOXO3A, upregulated the expression of Bax and P-CDK1, and downregulated the expression of Bcl-2 and Cyclin B1 (Zhang et al., 2021). Solasonine induced apoptosis by inhibiting the expression of the proteins p65 and Bcl-2, enhancing the expression of the proteins Bik and Bak, and activating the Caspase-3 pathway (Li et al., 2020). Zhang et al. showed that solamargine (**79**) induced apoptosis by decreasing the mitochondrial membrane potential, upregulating the expression of pro-apoptotic protein, and downregulating the expression of anti-apoptotic protein with an IC_50_ value of 9.81 μΜ (Zhang, 2018). In 2022, Yin et al. found that solamargine induces apoptosis and autophagy in liver cancer cells by regulating the LIF/miR-192-5p/CYR61/Akt signaling pathway at high doses, thereby inhibiting tumor cell proliferation. Solamargine at low doses regulates the biological function of immune cells by inhibiting the LIF/Stat3 signaling pathway, reshaping the tumor microenvironment, and inhibiting the process of tumor cell heterogeneity, thereby exerting a synergistic antitumor effect. This provides a scientific basis for its wide application in modern clinical treatment (Yin et al., 2022). In the clinic, adjuvant therapies, such as radiotherapy, endocrine therapy, chemotherapy, and targeted therapy, are administered to patients with advanced or metastatic tumors, but subsequent side effects usually result in treatment failure and increased mortality. Hence, the need for drugs with strong efficacy but minimal side effects and toxicity is great. Degalactotigonin (**2**) isolated from *S. nigrum* can inhibit the proliferation, invasion, migration and tumorigenicity of renal cell carcinoma cells. It can be used as an effective drug for the treatment of advanced renal cell carcinoma (Wang et al., 2020). This provides strong evidence that TCM can be better applied in the clinic.

### Immunomodulatory Activity

*S. nigrum* crude polysaccharides SNLP-1 displayed obvious immunoregulatory activity for macrophages by promoting the release of NO and the secretion of cytokines (TNF-α and IL-6) *in vitro*, and further mechanistic studies have indicated that SNLP-1 improves the gene and protein expression levels of TLR4 and its key nodes MyD88, TRAF-6, NF-κB, and c-JUN in the pathway. Establishing a lung cancer mice model and oral administration with 200 mg/kg/day SNLP-1, SNLP-1 not only reduced the tumor weight of lung cancer mice, but also increased the index of thymus and spleen. Flow cytometry revealed that the ratio of T lymphocyte subsets CD4^+^/CD8^+^ and the concentrations of serum Th1 cytokines (IFN-*γ*, IL-2, TNF) in mice were increased by SNLP-1. This indicated that the homogeneous polysaccharide fraction SNLP-1 can enhance the function of the body’s immune system *in vivo* and *in vitro* (Pu, 2020). Similarly, prepared and cultured mouse spleen lymphocytes with LPS and ConA, thymus and spleen index, B and T lymphocyte proliferative transformation, and NK cell activity were significantly higher in three groups than in the control group after daily oral administration of 800, 400 or 200 mg/kg crude polysaccharides of *S. nigrum* for 28 days (Tian et al., 2019). Compared with the control group, *S. nigrum* water extract polysaccharide (SNLWP-1) and alkali extract polysaccharide (SNLAP-1 and SNLAP-2) significantly increase the weight of immune organs and serum IL-2 and IFN-*γ* in H22 tumor bearing mice (Ding et al., 2012b). Other extractions protocols, including decoction also exhibited immunomodulatory activity in clinical studies. Treatment with commissioned Longkui Yinxiao Tablet for 8 weeks resulted in higher CD4^+^/CD8^+^ and CD4^+^ levels in the psoriasis treatment group and lower CD8^+^ and cytokines TNF-α, IL-6, and IL-17 levels compared with the control group (Dai et al., 2018).

The above literature indicated that *S. nigrum*, especially polysaccharides, has immunomodulatory activity *in vitro* and *in vivo.* However the immunomodulatory mechanism of *S. nigrum* is limited to the level of inflammatory factors, and there is a lack of in-depth exploration of its immunomodulatory mechanism. Research on effective immunomodulatory components mainly focuses on polysaccharides, but more attention should be paid to other effective components.

### Anti-Inflammatory Activity

**FIGURE 4:**
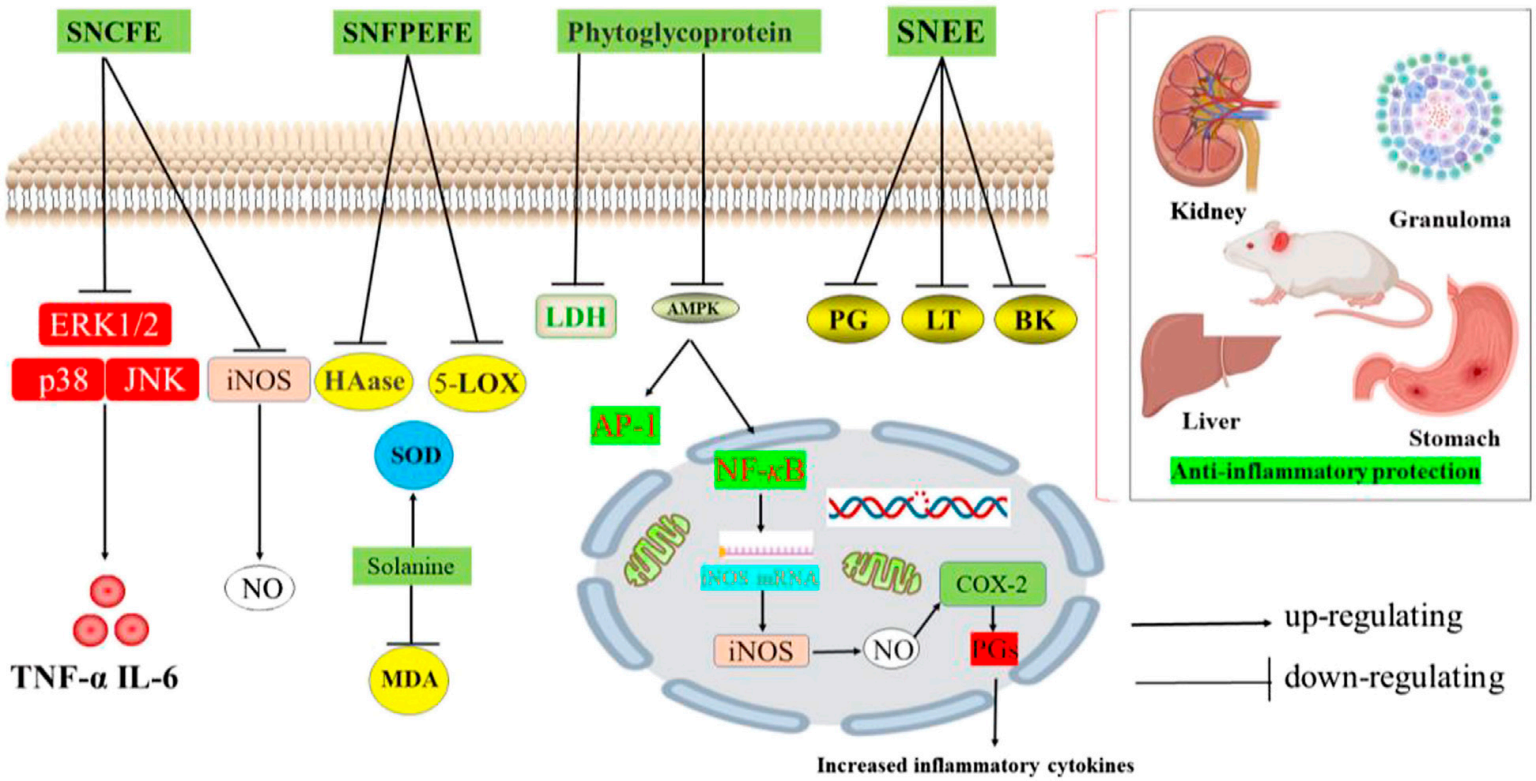
Schematic representation of the molecular mechanism of anti-inflammatory activities of crude extracts or isolated compounds from *S. nigrum*. (SNCFE, Chloroform fraction extracts of *S. nigrum*; SNFPEFE, Petroleum ether fraction extracts of *S. nigrum* fruit; SNEE, Ethanol extracts of *S. nigrum*).

Inflammation is the immune system’s response of the body to pathogenic factors and their damaging effects, and it is a self-protective response to help the body recover and resist infection, disease and pain. Relevant studies have confirmed the anti-inflammatory activity of crude extracts of *S. nigrum* in various inflammation related models and explained the possible related mechanisms, and a possible mechanism of action is shown in Figure 4. Chloroform extract of *S. nigrum* showed 80% inhibition of the NO and iNOS production stimulated by LPS at a concentration of 50 μg/ml. TNF-α and IL-6 are cytokines, which are responsible for the pathogenesis of many inflammatory disorders. Thus, the levels of TNF-α and IL-6 measured by ELISA on LPS-induced peritoneal macrophages have been determined, and the results showed that the chloroform fraction reduced the TNF-α and IL-6 levels in a dose-dependent manner and inhibited the phosphorylation of p38, JNK and ERK1/2, which may account for the anti-inflammatory mechanism of the *S. nigrum* chloroform fraction (Kang et al., 2011). Yeom et al. showed in the TPA-induced acute ear edema mouse model that the cell viability in fruit extract of *S. nigrum* in 80% ethanol was 51.35% at the concentration of 0.125 mg/ml. The NO production in *S. nigrum* fruit extract in 80% ethanol decreased to 4.1%, 16.1%, 61.0%, and 79.8% at concentrations of 0.125, 0.250, 0.500, and 1.000 mg/ml, respectively, and could also relieve edema and decrease the thickness of ear tissue *in vivo*, probably accounting for the abundance of alkaloid and flavonoid (Yeom et al., 2019). Similar results in acute and subacute rat models, and the hydroalcoholic extracts of *S. nigrum* showed less deposition of macrophages and more deposition of collagen fibres and exhibited organ protection properties (liver, stomach, and kidney), perhaps owing to the presence of steroidal alkaloids and steroidal saponins of *S. nigrum* (Aryaa and Viswanathswamy, 2017). Self-made *S. nigrum* suppository promoted the repair of damaged epithelial tissue and restored prostatic secretion through the decrease of the rat prostate wet quality and the white blood cell count and the increase in the density of lecithin corpuscle (Xu et al., 2015). Likewise, self-made *S. nigrum* could increase the SOD activity and decrease the MDA content to exhibit anti-inflammatory activity in the rabbit synovium after the rabbit knee joint was coated with self-made *S. nigrum* ointment for 1.5 h per day for 6 days in total in an electroacupuncture-made rabbit knee synovitis model (Li J et al., 2021). In 2017, Wang et al., isolated nine new steroidal saponins from berries of *S. nigrum*, Among these steroidal saponins, solanigrosides Y1 (**52**) significantly inhibited the NO production with an IC_50_ value of 9.7 μM, and some compounds exhibited significant inhibitory effects on the LPS-induced IL-6 and IL-1*β* production (Wang et al., 2017). In 2018, Xiang et al. isolated seven previously undescribed steroidal glycosides from the unripe berries of *S. nigrum*, and all seven compounds exhibited inhibitory activities on the NO production with IC_50_ values ranging from 11.33 to 49.35 µM (Xiang et al., 2018). Overall, the extracts and other preparations from *S. nigrum* deserve more studies related to inflammatory diseases.

### Antibacterial and Larvicidal Activity

So far, bacterial and fungal infections and multidrug resistance have been great threats to human health and are important challenges that need to be urgently solved. Pharmacological studies have demonstrated that the antifungal activities of different parts of *S. nigrum* prepared in four solvents (ethanol, chloroform, petroleum ether, and distilled water) to struggle against five fungal strains viz. *Aspergillus’s Niger*, *A. flavors*, *Saccharomyces cervisae*, *Alternaria alternate*, and *Fusarium oxysprum*. The MIC was found to be in the range of 250–1,000 μg/ml, and the zone of inhibition was measured in the range of 9.3 mm of stem extract in distilled water against *A. flavus* and 32.42 mm of fruit extract in chloroform against *A. niger* (Mazher et al., 2017). The maximum zones of inhibition of ethanol extract of *S. nigrum* were 16.88, 11.33, and 19.25 mm for *Staphylococcus aureus*, *Escherichia coli*, and *Aeromona sobria* at a concentration of 200 mg/ml, respectively (Ge, 2019). The isolated compound, solasodine-3-*O-*β-d-glucopyranoside (**93**) displayed potent fungicidal activity against both *azole-sensitive* and *azole-resistant Candida albicans* strains in spider medium with the MIC value of 32 mg/ml, which may be attributed to the essential role of the glucosyl moiety. Subsequent pharmacological mechanism studies found that solasodine-3-*O*-*β*-d-glucopyranoside alkalized the intracellular vacuole and elicited vacuole permeability to contribute to cell death in *C. albicans* (Chang et al., 2017). Another study showed that solasodine-3-*O-β*-d-glucopyranoside could attenuate the virulence of *Candida albicans* through inhibiting adhesion and yeast-to-hyphal morphological transformation, and the results of the XTT reduction assay showed that solasodine-3-*O-β*-d-glucopyranoside could inhibit biofilm formation of *C. albicans* at concentrations of 16 mg/L or above. Next, downregulation of adhesion-related genes such as ALS3, EAP1, and HWP1 with the addition of 1 mM cAMP rescued the growth rate of solasodine-3-*O-β*-d-glucopyranoside-treated biofilm from 27.11% to 77.15%. The results suggested that solasodine-3-*O-β*-d-glucopyranoside inhibits the activity of *C. albicans* via regulating the Ras-cAMP-PKA signaling pathway and reducing the intracellular cAMP content (Li et al., 2015).

After exposition to graded concentrations (2.5, 5, and 10 ppm) of the synthesized silver nanoparticles (AgNPs) to fight against third instar larvae of *C.quinquefasciatus* and *An. Stephensi*, the IC_50_ and IC_90_ values of dry leaf, fresh leaf, and berry extracts of *S. nigrum* for *An. Stephensi* were 1.33, 1.59, and 1.56 ppm and 3.97, 7.31, and 4.76 ppm, respectively. The IC_50_ and IC_90_ values of dry leaf, fresh leaf and berry extracts of *S. nigrum* for *C.quinquefasciatus* were 1.26, 1.33, and 2.44 ppm and 14.38, 38.04, and 13.42 ppm, respectively (Rawani et al., 2013). To explore the acaricidal activity of crude extracts of *S. nigrum* for *Tetranychus cinnabarinus*, Chen et al. conducted slide-dip and leaf-dip assays and glasshouse experiments, revealing LC_50_ and LC_90_ values of 3.50, 3.99, 4.70, 12.15, 13.99, and 14.92 g/L after 1, 3, and 7 days of treatment, respectively. (Chen and Dai, 2016). The methanol leaf extract possessed the highest larvicidal activity (mortality rate of 90%) against the early fourth-instar larvae of *C. quinquefasciatus* at 1,000 ppm (Rahuman et al., 2009). Leaf extract from *S. nigrum* caused mortality rates of 28.54%, 56.8%, and 57.42% of the green peach aphid after exposure for 24, 48, and 72 h (Madanat et al., 2016). In addition, the active compounds solamargine and solasonine isolated from *S. nigrum* were found to fight against *Plasmodium yoelii* 17XL in mice, and the parasitemia suppressions of solamargine and solasonine were 64.89% and 57.47%, respectively (Chen et al., 2010). However, there is a lack of research on the mechanism underlying the insecticidal effect.

### Antioxidant Activity

Excessive accumulation of free radicals in the body may cause aging and tissue damage. Oxidative stress is the pathogen of many human diseases, such as atherosclerosis, ischemic reperfusion injury, inflammation, cancer, aging, and neurodegenerative diseases. Antioxidants are the only weapon against the oxidizing and damaging effects of free radicals on the body’s vital chemicals and cells. Various studies focus on the antioxidant activity of the bioactive compounds or extracts of *S. nigrum* using *in vitro* and *in vivo* assays. Sivaraj et al. carried out DPPH˙ radical, superoxide radical, ABTS˙^+^ radical cation, phosphomolybdenum reduction, and Fe^3+^ reducing power assays of the fruit extract of *S. nigrum*. The maximum DPPH˙ radical scavenging activity was 73.16% at 120 µg/ml (IC_50_ = 81.02 μg/ml), the maximum superoxide radical scavenging activity was 60.06% at 120 μg/ml (IC_50_ = 57.82 μg/ml), the maximum ABTS˙^+^ radical cation scavenging activity was 67.70% at 60 µg/ml (IC_50_ = 35.56 μg/ml), the maximum phosphomolybdenum reduction was 96.77% at 120 µg/ml (RC_50_ = 21.25 μg/ml), and the maximum Fe^3+^ reduction was 72.10% at 120 µg/ml (RC_50_ = 63.74 μg/ml). These results indicated that two compounds, flavone and oleic acid, may be one of the reasons for the antioxidant property of the fruit extract of *S. nigrum* (Sivaraj et al., 2020). GSH and ROS levels determined in primary rat astroglial cell cultures showed that the leaf extract of *S. nigrum* could increase intracellular GSH levels and decrease ROS levels, which indicated that the leaf extract of *S. nigrum*. could restore the oxidative status by using glutamate as a stressor, prevent the increase in the glutamate uptake, and inhibit the excitotoxicity of glutamate (Campisi et al., 2019). According to the hydroxyl radical scavenging assay, 60% ethanol crude extract of *S. nigrum* had DPPH scavenging a rates of 68.45% and 49.12% (Teng et al., 2014). Lunasin peptide, purified from *S. nigrum*, inhibited oxidative DNA damage in a dose dependent manner by blocking the Fenton reaction between Fe^2+^ and H_2_O_2_ by chelating Fe^2+^ suggesting that lunasin peptide may play an important role in the chemoprevention for oxidative carcinogenesis (Jeong et al., 2010). After the treatment with glucose oxidase or xanthine oxidase alone or in combination with 0.01, 0.1, 1, 10, and 20 μg/ml of glycoprotein for 4 h, the viabilities of the NIH 3T3 cells were 40.4%, 47.3%, 53.0%, 68.2%, and 85.3%, respectively, compared with 33.3% of the control group (glucose oxidase). The viabilities were 75.1%, 79.3%, 83.2%, 86.0%, and 95.1%, respectively, compared with 71.2% of the control group (xanthine oxidase) (Heo and Lim, 2004). Overall, the extracts and compounds from *S. nigrum* may be suitable antioxidants for the further investigations.

### Hepatoprotective Activity

The water extract of *S. nigrum* (SNWE) demonstrated the potential to protect the liver against diseases. Administration of SNWE at dosages of 0.2, 0.5, and 1.0 g/kg for 6 weeks in CCl_4_-induced chronic hepatotoxic rats reversed the body and organ weights and caused cloudy swelling, necrosis, cytoplasmic vacuolization and fatty degeneration determined by both qualitative and quantitative histopathological examinations. Furthermore, SNWE also reduced the levels of serum liver enzyme markers (GOT, GPT, ALP, and total bilirubin), superoxide and hydroxyl radicals and restored the GSH and SOD contents to the normal level especially at high doses of 0.5 and 1.0 g/kg (Lin et al., 2008). In AAF-induced liver damage rats, the liver/body weight ratios were 3.1- and 2.9-fold of that of the control group for 1% and 2% SNWE supplement, respectively. SNWE also decreased the levels of the serum biomarkers GOT, GPT, *γ*-GT, and AFP and induced the expression and the activation of both GSTs (GST-α, and GST-μ), which were responsible for the metabolism of a broad range of xenobiotics and carcinogens. Moreover, the treatment of SNWE regulated the level of Nrf2 and the level of downstream antioxidant enzymes regulated by Nrf2, including GPx, SOD-1, and catalase (Hsu et al., 2009). Chester et al. reported that the hydroalcoholic extract (250 mg/kg) of *S. nigrum* showed a significant decrease in hepatic GSH, SOD, and CAT and considered this as an index of the antioxidant status of tissues in d-galactosamine-induced hepatic fibrosis rats. Histopathological study also showed that the crude extract had a protective effect on the liver due to the antioxidant properties of the plant (Chester et al., 2019). Polysaccharides, extracted from *S. nigrum*, alleviated liver swelling, increased the levels of SOD, GSH, and CAT, decreased the content of MDA (Yang et al., 2014).

### Neuroprotective Activity

With the development of modern medicine, there are increasing investigations to illustrate the mechanisms of the bioactive constituents isolated from *S. nigrum*, promoting the research and applications in clinic in turn. Regarding the central nervous system, Ogunsuyi et al. investigated the neuroprotective effect of *S. nigrum* on an oscopolamine-induced cognitive impairment model in rats. Pretreatment with 10% *S. nigrum* inclusions could also significantly restore the impaired memory function, decrease the AChE activity, MDA content, and BChE activity, and increase the brain GSH content (Ogunsuyi et al., 2018). In 2019, Ogunsuyi et al. continued to study the neuroprotective effect of *S. nigrum* in *Drosophila melanogaster*, and the results also showed that the impaired behavioral physiology and enzyme activities (glutathione-S-transferase, monoamine oxidase, cholinesterase) were ameliorated in Al-treated flies after the daily administration of pulverized vegetables for 7 days (Ogunsuyi et al., 2020). In 2021, Ogunsuyi et al. proved that 1% dietary inclusions of *S. nigrum* could decrease the survival rate and ROS levels and increase the total thiol contents *in vivo* (Ogunsuyi et al., 2021).

### Anticholesterol Activity

Polyphenols derived from *S. nigrum* were reported to be an antiobesity agent, which could promote hepatic lipolysis, decrease serum triacylglyceride, cholesterol, and low-density lipoprotein (LDL)-cholesterol and inhibit lipogenesis (Peng et al., 2020). In addition, the oral administration of an ethanolic extract or chloroform fraction of *S. nigrum* (200 and 400 mg/kg) for 5 days to triton-induced hyperlipidemic rats reversed the elevation of the serum of total cholesterol, triglycerides and LDL cholesterol level and the reduction of the HDL cholesterol level (Sharma et al., 2012).

## Clinical Effectiveness in Humans

As a folk medicine widely used around the world, *S. nigrum* has been widely reported clinically in recent years. The application of decoction of the whole herb or fruit of *S. nigrum* and its compound preparations for the treatment of liver cancer, lung cancer, cervical cancer, esophageal cancer, breast cancer, nasopharyngeal cancer, and other malignant tumors has attracted the attention of scholars at home and abroad for its remarkable clinical efficacy (Mei et al., 2011).

Liver cancer is currently the fourth most common malignant tumor and the second leading cause of cancer death in China, which seriously threatens the life and health of the Chinese people. In an open, prospective, randomized clinical trial conducted from 2012 to 2015, the clinical efficacy of *S. nigrum* tablets in the treatment of liver cancer was evaluated. Eighty-two patients with liver cancer were divided into observation group and control group according to the random number table method. The patients in the control group were treated with sorafenib, and the patients in the observation group were treated with *S. nigrum* tablets on the basis of the control group. The clinical efficacy, liver function recovery, inflammatory factor levels and survival rate were compared between the two groups. Results: The complete remission rate and total effective rate in the observation group were 14.63% (6/41) and 43.90% (18/41), which were significantly higher than those in the control group (2.44% (1/41) and 14.63% (6/41); the number of patients with liver function recovery in the observation group at 3 months, 6 months, and 1 year of treatment were significantly higher than those in the control group; after treatment, the IL-1, IL-6 and TNF-α levels were significantly lower than those in the control group. The 1-year survival rate and 2-year survival rate of the observation group were significantly higher than those of the control group. These research results show that *S. nigrum* tablet has a significant effect in the treatment of liver cancer, can effectively promote the recovery of liver function, improve the level of inflammatory factors, and improve the survival rate of patients (Yang et al., 2017). In addition, another clinical experimental study showed that the treatment of patients with advanced primary liver cancer with *S. nigrum* mixture can improve the clinical symptoms, liver function and immune function of the patients, effectively improve the quality of life of the patients, and is expected to prolong the survival period (Huang and Guan, 2013). In view of the diverse *in vitro* and *in vivo* pharmacological activities of S. nigrum, larger-scale randomized, double-blind, and controlled trials are needed to verify its clinical efficacy.

## Toxicity

Although *S. nigrum* has been used as a drug or food for thousands of years and a large number of pharmacological activities have been reported, there are limited reports on the safety and side effects of the plant and its bioactive components. In 2005, Lai et al. studied the acute toxicity and genotoxicity assay of *S. nigrum*. The highest gavage dose of 21.5 g/kg was administered to mice, and no signs of toxicity and death were observed and recorded for 14 days of continuous gavage. In the genotoxicity test, the short-term mutagenicity of *S. nigrum* juice was investigated by the mouse sperm deformation test, micronucleus test, and Ames test. The results of these three tests were all negative, indicating no genotoxic effect (Lai et al., 2005). In 2014, Mo et al. experimentally confirmed the maximum dose of 494.4 g/kg of aqueous decoction of *S. nigrum* in mice. When normal people take the commonly used amount of *S. nigrum* (30–60 g), toxic and side effects are rarely observed. However, overdose can cause poisoning, resulting in symptoms such as headache, abdominal pain, vomiting, diarrhea, pupil dilation, arrhythmia, coma, and other symptoms (Mo et al., 2014).

Modern research showed that *S. nigrum* mainly contains active chemical ingredients such as alkaloids, saponins, and polysaccharides. Among these active ingredients, steroidal alkaloids have antitumor, liver and kidney protective, antipyretic, analgesic, anti-inflammatory, and expectorant effects. The steroidal alkaloids in *S. nigrum* not only have a wide range of pharmacological activities but also have some toxicity. Representative compounds are solasonine and solamargine, both of which are steroidal alkaloids composed of solanidine as an aglycon. Solanine causes strong irritation to gastrointestinal mucosa (Li, 2019). In addition, other toxicological tests have also confirmed that solanine can affect the embryonic development, causing miscarriage and stillbirth. The results of solanine on the bone marrow cell cycle of male mice showed that the ratio of cells in G0/G1 phase increased with increasing dose, while the ratios of S phase and G2/M phase decreased, and cells were blocked in G0/G1 phase. Affect the synthesis of DNA, and then affect the G2/M phase, so that the number of cells entering this phase is reduced. The incidence of micronucleus and sperm deformity increased gradually with the dose. Therefore it has potential mutagenic effects and certain genetic toxicity (Ji et al., 2009). However, the content of solanine in the leaves, stems and fruits of *S. nigrum* will gradually decrease as the plant grows. Preliminary toxicological studies of *S. nigrum* showed less toxicity and a certain impact on the liver and kidney function. In the future, a large amount of clinical and animal data will be needed to verify the safety of *S. nigrum* for better use as medicine in the field of clinical and health care and in food homologous products.

## Conclusion and Future Perspectives

This review systematically summarizes the latest findings on the botany, traditional uses, phytochemistry, pharmacology, clinical trials, and toxicity of *S. nigrum.* Regarding the use as a medicinal and edible plant, there are records in the dietary histories of China and India that the leaves and fruits of *S. nigrum* were cooked as food. *S. nigrum* has been used to treat various cancers, diarrhea, fever, itchy skin, dysentery, edema, and other diseases in the indigenous peoples of China, India, Italy, Turkey, Yemen, Jordan, and Libya for more than a thousand years. Regarding the research on the phytochemical constituents of *S. nigrum*, 188 small molecule compounds and dozens of polysaccharides have been isolated and identified. Through systematic analysis, polysaccharides, steroidal saponins, and alkaloids were identified as the representative active components of *S. nigrum* with numerous pharmacological activities, which have been demonstrated in *in vitro* and *in vivo* investigations. Steroidal alkaloids represented by compounds **79** and **100** and polysaccharides represented by SNL-P1a have been considered to be biologically active components with extensive biological properties, including antitumor, anti-inflammatory, immunomodulatory, antimalarial, antibacterial, and antiviral effects. A large number of pharmacological studies have shown that **79** is a very promising candidate for the treatment of cancer. The content of vitamin C in the *S. nigrum* fruit is quite high, which has good nutritional and health value. The antitumor effect of *S. nigrum* polysaccharide is mainly through improving the immunity of the body. To sum up, as a food and medicinal resource, *S. nigrum* has a good health care function and important edible and medicinal value, and deserves more development and research.

However, a number of points also need to be improved: 1) A large number of studies on the *in vitro* and *in vivo* activities of *S. nigrum* extracts are listed in Table 3, but the material basis for the pharmacological activities of these crude extracts is still unknown. A large number of compounds have been isolated from *S. nigrum*, but current research on these compounds may be just the tip of the iceberg. Research on the chemical composition of *S. nigrum* mainly focuses on saponins, alkaloids, and polysaccharides, while the research on organic compounds, such as phenolic acids represented by flavonoids, is relatively rare. Therefore, research aimed at clarifying the active ingredients in *S. nigrum*, and the mechanism of action of the active ingredients should be further elucidated. For the trace active components in *S. nigrum*, its structure and activity mechanism can be studied through new technologies such as efficient preparation, computer virtual screening, and target fishing. 2) The composition and content of steroidal saponins and steroidal alkaloids in *S. nigrum* will change with the plant growth. To further explore the material basis of its active components, the composition and content of *S. nigrum* at different growth stages should be identified, and the biosynthetic pathway should be explored. 3) Toxicological studies are critical to assess the safety of herbal medicines, but data on the toxicology of *S. nigrum* remain scarce. It has been reported that the unripe *S. nigrum* fruit has a certain toxicity and can cause human poisoning and harm to human health. Therefore, it is necessary to carry out systematic toxicity and safety assessment studies on *S. nigrum* extracts and active ingredients to ensure full utilization of drug resources, meet Western standards of evidence-based medicine, and provide accurate evidence for clinical application. By studying the metabolites and metabolic mechanisms of drugs in the body, new drugs with higher biological activity and safer can be found. Therefore, pharmacokinetic, pharmacodynamic, and toxicological research are equally important in the process of drug development. Research on the drug metabolism of *S. nigrum* should be increased. 4) Since the *S. nigrum* plant contains potentially toxic compounds such as compound **79**, reliable analytical methods are required for proper quality control of product development to ensure that potentially toxic components in *S. nigrum* products are kept below tolerated levels. 5) *S. nigrum* fruit is rich in vitamin C and polysaccharides with immunomodulatory effects. The processed products of *S. nigrum* need further research, exploration, and utilization, especially as a formula for fruit juice, fruit wine, and cosmetics.

Taken together, *S. nigrum* has attracted great interest as a medicinal and edible herb because of its rich nutrition and wide range of pharmacological activities. It has great development potential in the fields of functional food and TCM, and also provides resources for the lead compound library in the process of new drug development. However, in addition to providing opportunities, the application of *S. nigrum* also exhibits challenges. Facing the weak links and specific problems of the development of TCM, more time and research efforts need to be devoted to the high-quality development of TCM. We believe that this review can provide a valuable reference for the future development and utilization of *S. nigrum*.
